# Textured Perovskite/Silicon Tandem Solar Cells Achieving Over 30% Efficiency Promoted by 4-Fluorobenzylamine Hydroiodide

**DOI:** 10.1007/s40820-024-01406-4

**Published:** 2024-05-02

**Authors:** Jingjing Liu, Biao Shi, Qiaojing Xu, Yucheng Li, Yuxiang Li, Pengfei Liu, Zetong SunLi, Xuejiao Wang, Cong Sun, Wei Han, Diannan Li, Sanlong Wang, Dekun Zhang, Guangwu Li, Xiaona Du, Ying Zhao, Xiaodan Zhang

**Affiliations:** 1 Renewable Energy Conversion and Storage Center, Solar Energy Conversion Center, Institute of Photoelectronic Thin Film Devices and Technology, Nankai University, Tianjin, 300350 People’s Republic of China; 2 Key Laboratory of Photoelectronic Thin Film Devices and Technology of Tianjin, Tianjin, 300350 People’s Republic of China; 3 Haihe Laboratory of Sustainable Chemical Transformations, Tianjin, 300192 People’s Republic of China; 4 Engineering Research Center of Thin Film Photoelectronic Technology of Ministry of Education, Tianjin, 300350 People’s Republic of China; 5 Collaborative Innovation Center of Chemical Science and Engineering (Tianjin), Tianjin, 300072 People’s Republic of China; 6 Center of Single-Molecule Sciences, Institute of Modern Optics, Tianjin Key Laboratory of Micro-Scale Optical Information Science and Technology, College of Electronic Information and Optical Engineering, Nankai University, 38 Tongyan Road, Jinnan District, Tianjin, 300350 People’s Republic of China; 7 Shenzhen Research Institute of Nankai University, 16Th Floor, Yantian Science and Technology Building, Haishan Street, Yantian District, Shenzhen, 518083 People’s Republic of China

**Keywords:** Perovskite crystallization, (111) preferred orientation, Defect passivation, Perovskite/silicon tandem solar cells

## Abstract

Monolithic textured perovskite/silicon tandem solar cells (TSCs) are expected to achieve maximum light capture at the lowest cost, potentially exhibiting the best power conversion efficiency. However, it is challenging to fabricate high-quality perovskite films and preferred crystal orientation on commercially textured silicon substrates with micrometer-size pyramids. Here, we introduced a bulky organic molecule (4-fluorobenzylamine hydroiodide (F-PMAI)) as a perovskite additive. It is found that F-PMAI can retard the crystallization process of perovskite film through hydrogen bond interaction between F^−^ and FA^+^ and reduce (111) facet surface energy due to enhanced adsorption energy of F-PMAI on the (111) facet. Besides, the bulky molecular is extruded to the bottom and top of perovskite film after crystal growth, which can passivate interface defects through strong interaction between F-PMA^+^ and undercoordinated Pb^2+^/I^−^. As a result, the additive facilitates the formation of large perovskite grains and (111) preferred orientation with a reduced trap-state density, thereby promoting charge carrier transportation, and enhancing device performance and stability. The perovskite/silicon TSCs achieved a champion efficiency of 30.05% based on a silicon thin film tunneling junction. In addition, the devices exhibit excellent long-term thermal and light stability without encapsulation. This work provides an effective strategy for achieving efficient and stable TSCs.

**Supplementary Information:**

The online version contains supplementary material available at 10.1007/s40820-024-01406-4.

## Highlights

- Evaporation and solution two-step hybrid process are used to fabricate conformal growth of perovskite film on textured silicon substrates.
- F-PMAI additive enlarged the perovskite grains, induced the (111) preferred orientation and passivated interface defects.
- We obtained a champion efficiency of 30.05% for a monolithic perovskite/silicon tandem solar cell based on a silicon thin film tunneling junction.

**Supplementary Information:**

The online version contains supplementary material available at 10.1007/s40820-024-01406-4.

## Graphical abstract

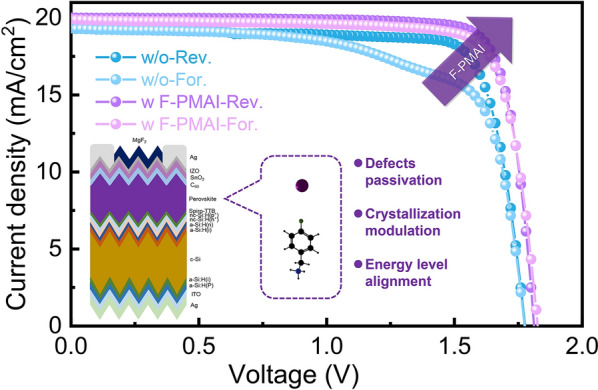

## Introduction

Currently, crystalline silicon (c-Si) solar cells still dominate the solar photovoltaic market, with recent research pushing their efficiency to 26.81% [1], approaching its theoretical limit power conversion efficiency (PCE) of 29.4% [2]. To further improve efficiency, multi-junction solar cells are considered to be one of the most promising ways to overcome the limit efficiency of single-junction cells by reducing thermalization loss [3, 4]. Particularly, the monolithic perovskite/silicon tandem solar cells (TSCs) have emerged as a key area of focus due to their potential for higher efficiency at lower costs [5–7] and have recently achieved a breakthrough PCE of up to 33.9% [1]. The most efficient designs of these TSCs are based on silicon substrates that are either polished or sub-microtextured on the front side to ensure compatibility with solution-processed perovskite films [8–10]. Despite their advancements, these cells still face challenges in light capture efficiency due to significant surface reflection [11–13], and the requisite chemical or mechanical polishing processes add considerable manufacturing costs [14, 15]. Consequently, people gradually focus on developing perovskite/silicon TSCs with industrially feasible textured silicon bottom cells. Both the theoretical insights [11] and experimental data [16] have demonstrated that conformal deposition on textured silicon cells has significant optical advantages. Employing a hybrid evaporation-solution process for this deposition has proven to be an effective method, and producing more efficient perovskite/silicon TSCs [16–19].

Numerous studies have been dedicated to optimizing the grain size, crystallinity and morphology of perovskite films to improve device efficiency by reducing grain boundaries and related defects [20, 21]. The evaporation-solution two-step process can realize the conformal growth of perovskite film on textured silicon substrates. Yet, compared to conventional all-solution processing methods used on polishing or sub-microtextured substrates, the vacuum deposition of inorganic components leads to narrower composition tunability and less crystallization controllable of perovskites [22–24]. Furthermore, tandem solar cells still face stability challenges. Perovskite crystals are known for their pronounced anisotropic photoelectric properties, in their energy levels, carrier lifetimes, and mobilities [25, 26]. Notably, the (111) facet of FAPbI_3_ films has been shown to significantly improve the stability of PSCs due to its advantageous carrier mobility, low exciton binding energy and thermodynamic stability [26–28]. However, research on the preferred (111) crystal orientation in textured perovskite/silicon tandem solar cells (TSCs) is lacking. The hybrid two-step perovskite crystallization process, which involves the infiltration of organic salts into a thermally evaporated inorganic framework followed by a self-assembly reaction to form the perovskite structure, often leads to incomplete reactions [22]. This can result in the accumulation of interfacial carriers and the formation of defects within the perovskite films, causing significant photocurrent hysteresis and constraining the performance of devices, especially those based on textured silicon substrates. Therefore, it is crucial to explore strategies for controlling the crystallization orientation and passivating interface defects on TSCs based on industry-relevant textured silicon substrates, further promoting the efficiency and stability of perovskite/silicon TSCs.

In this work, we introduced 4-fluorobenzylamine hydroiodide (F-PMAI) to the organic salts to fabricate conformal high-quality perovskite films by a hybrid evaporation-solution process. We have found that F-PMA^+^ can passivate the uncoordinated Pb^2+^/I^−^ while F^−^ can form hydrogen bonds with organic cations, thereby retarding the perovskite crystal growth, enlarging grain size and reducing film defects. Moreover, the F-PMAI molecule can decrease surface energy of the (111) facet facilitating the growth of (111) plane and the molecules are extruded from the perovskite to the bottom and top surface of perovskite film after growth, which leads to improved charge carrier transport and interface passivation, achieves excellent optoelectronic properties and enhanced stability on the single-junction solar cells and perovskite/silicon TSCs based on commercially textured silicon substrates. The champion 1.60 eV single-junction cell with an efficiency of 22.74% indicated a high fill factor (*FF*) of 85% and an improved open-circuit voltage (*V*_OC_) of 1.15 V. When F-PMAI was applied to the top perovskite cell for a monolithic perovskite/silicon TSC, a champion PCE of 30.05% with reduced hysteresis was obtained. In particular, the unencapsulated tandem device showed excellent thermal stability (maintained 90% of initial PCE after 1650 h under 85 °C N_2_ glovebox) and light stability (700 h, no decay).

## Experimental Section

### Materials

All materials were purchased and used without undergoing any further purification process. 2,2′,7,7′-tetra(N,N-di-tolyl)amino-9,9-spiro-bifluorene (Spiro-TTB) and C_60_ were purchased from Luminesce Technology Corp. Lead(II) iodide (PbI_2_) was purchased from Sigma-Aldrich. Cesium chloride (CsCl), formamidine bromide (FABr), formamidine iodide (FAI), methylamine hydrochloride (MACl) and 4-fluorobenzylamine hydroiodide (F-PMAI) 2,9-dimethyl-4,7-diphenyl-1,10-phenanthroline (BCP) were purchased from Xi’an Polymer Light Technology in China. Ethanol (99.5% anhydrous) and isopropyl alcohol (IPA) (99.5% anhydrous) were purchased from Alfa Aesar.

### Device Fabrication

#### Organic Salt Solution

For organic slat solution without additive: 0.14 mol FABr, 0.68 mol FAI and 0.1 mol MACl were dissolved in 1 mL ethanol. For the solution with F-PMAI: 15 mmol F-PMAI is additionally added to dissolve in the organic salt solution.

#### Fabrication of Single-Junction Perovskite Solar Cells

The single-junction devices were fabricated with the structure: ITO/Spiro-TTB/perovskite/C_60_/BCP/Ag. The patterned ITO glass substrates were sequentially cleaned with deionized water, acetone, and isopropanol in ultrasonic baths for 30 min, respectively. After pre-treating the cleaned ITO substrates under UV/O_3_ for 20 min, 8 nm of spiro-TTB was thermally evaporated at a rate of 0.1 Å s^−1^ as measured by a quartz crystal monitor at a working pressure < 6.5 × 10^−6^ mbar in a Lesker mini Spectros system. Then the 5 nm CsCl thin layer was evaporated at a rate of 0.4 Å s^−1^ to optimize energy level alignment. For the fabrication of perovskite film, first, the PbI_2_ and CsCl were co-evaporated at a rate ratio of 13.35:0.665 Å s^−1^ in the same chamber to form inorganic precursor film. Following, the organic salt solution without and with additives was dynamically spin-coated at a speed of 9,500 rpm for 30 s in the N_2_ glovebox, then annealed at 130 °C for 20 min in ambient air with a 30%–35% humidity. Subsequently, C_60_ and BCP were thermal evaporation at a rate of 0.7 Å s^−1^ for 23 nm and 0.2 Å s^−1^ for 8 nm, respectively. Finally, 80 nm of silver was thermally evaporated through a shadow mask to create the metal electrode. The active area was 0.08875 cm^2^. The preparation processes schematic diagram is shown in Fig. S1.

#### Fabrication of Silicon Bottom Solar Cells

The silicon heterojunction (SHJ) bottom cells were fabricated using double-side-textured n-type silicon wafers with a pyramid size of 3–5 µm. Before loading into the vacuum chamber, 1% HF solution is used to remove the oxide layer on the wafer surface and partially saturate the hanging bond on the wafer surface. The intrinsic passivation layer and doping layer of silicon heterojunction cells were prepared by plasma enhanced chemical vapor deposition (PECVD). Firstly, the silicon wafer was heated to ~ 170 °C in a vacuum chamber, and the intrinsic passivation layer and n-type doping layer were deposited on one side, then the silicon wafer was turned over, and the intrinsic passivation layer and p-type doping layer were deposited on the other side. In the deposition of a-Si:H or nc-Si:H, the mixture of SiH_4_ and H_2_ was used as the reaction gas. Phosphane is used as n-type doped phosphorus source and trimethyl boron or ethyl borane as p-type doped boron source. Following that, an n-type/p-type nc-Si: H layer stack was deposited successively on the front side in the same PECVD reactor, serving as the tunneling recombination junction.

#### Fabrication of Perovskite/Silicon Tandem Solar Cells

The preparation process of Spiro-TTB is the same as single junction. The thickness of the CsCl thin layer and the perovskite film is correspondingly increased by 1.5 times when deposited on the textured silicon bottom cells. Twenty-three nanometers of the C_60_ layer was thermally evaporated on the as-prepared perovskite film at a rate of 0.2 Å s^−1^. Then, the films were transferred to an atomic layer deposition (ALD) system (Veeco Savannah S200) where 20 nm of SnO_2_ was deposited at 100 °C using tetrakis (dimethylamino) tin (IV) and H_2_O_2_ as precursors. Afterward, an indium zinc oxide (IZO) transparent electrode (110 nm) was sputtered using a target (90% In_2_O_3_ + 10% ZnO) with a radiofrequency power of 38 W (sheet resistance of 40 Ω sq^−1^) in a Lesker sputtering system. Subsequently, thermal evaporation of 1 μm Ag through a shadow mask formed the front metal grids with 0.5003 cm^2^ active area and formed the back metal electrode with 1 cm × 1 cm area, respectively. Finally, an antireflective layer of MgF_2_ (100 nm) was evaporated at a working pressure of < 8 × 10^−6^ mbar at a rate of 2 Å s^−1^ in Alfa Aesar chamber. The preparation processes schematic diagram is shown in Fig. S2.

### Characterization

The current density–voltage (*J–V*) curves of single-junction perovskite solar cells and TSCs were measured using a Keithley 2400 Source meter, under AM 1.5G illumination (100 mW cm^−2^) with a xenon-lamp-based solar simulator (Enli. Tec., Taiwan). The area of the cells was determined by the aperture shade masks. The devices were measured at a scan rate of 20 mV s^−1^ and a delay time of 20 ms. For single-junction cells, the bias scan was from 1.2 to 0 V for reverse scan and from 0 to 1.2 V for forward scan. For tandem cells, the bias scan was from 2 to 0 V for reverse scan, and from 0 to 2 V for forward scan. The external quantum efficiency spectra (EQE) were measured by the Enli Tec (Taiwan) system. Space charge limited current (SCLC) measurement was taken in the dark at a scan rate of 20 mV s^−1^ with a delay time of 20 ms. The bias scan ranged from 0 to 5 V. Electrical impedance spectroscopy (EIS) measurement was taken by Metrohm Autolab (PGSTAT204). Mott–Schottky plot was obtained by Metrohm Autolab (PGSTAT204). The scanning electron microscopy (SEM) images of top views and cross sections were measured by FEINanoSEM650. X-ray diffraction (XRD) was measured by a multi-functional diffractometer (Rigaku, ATX-XRD) with Cu Kα radiation (λ = 1.5405 Å). X-ray photoelectron spectrometry (XPS) and ultraviolet photoelectron spectrometry (UPS) analysis were conducted with a photoelectron spectrometer (Thermo Scientific ESCALAB250Xi, UK). Photoluminescence (PL) and time-resolved photoluminescence (TRPL) spectroscopy were measured by a PL spectrometer FLS980 (Edinburgh Instruments), and a pulsed laser with a wavelength of 475 nm was used as the excitation source. Kelvin probe force microscopy (KPFM) and conductive atomic force microscopy (C-AFM) measurements were taken by Bruker Dimension Icon. The Fourier transform infrared (FTIR) was measured by Thermo Nicolet Nexus intelligent Fourier transform infrared spectrometer. Transient photovoltage (TPV) decay were recorded in an electrochemical workstation (IM6ex, Zahner, Germany) under ambient condition. The contact angles of the samples were performed with an automatic contact angle measuring apparatus (Dataphysics OCA-20, Germany). The time-of-flight secondary ion mass spectroscopy (ToF–SIMS) was measured by ToF–SIMS 5 in Germany GmbH. The PL mapping measurements were taken by using a Q2 multi-dimensional confocal fluorescence microscopy imaging system, equipped with an excitation wavelength 488 nm. The grazing-incidence wide-angle X-ray scattering (GIWAXS) was measured by Xeuss 2.0, using a copper target with a light tube power of 30 W and wavelength of 1.5 Å.

## Results and Discussion

### Effect of F-PMAI Incorporation on the Surface Chemistry and Crystal Quality of Perovskite Films

**Fig. 1:**
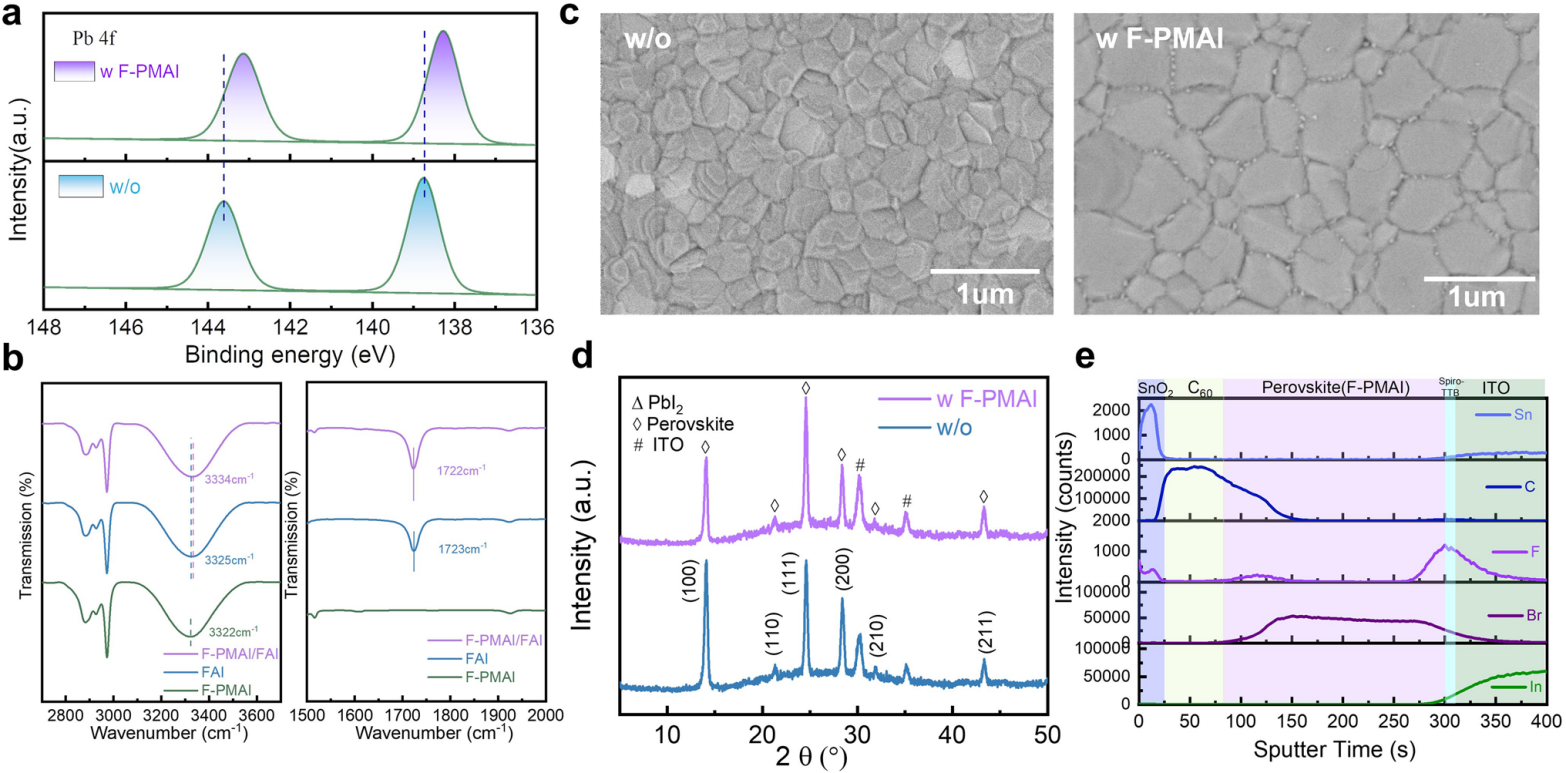
Surface chemistry and crystal quality of perovskite films. **a** XPS spectra of Pb 4*f* of perovskite films without and with F-PMAI additive. **b** Local magnification of FTIR spectra of F-PMAI, FAI and FAI/F-PMAI mixture. **c** Top-view SEM images of final perovskite films without and with F-PMAI. **d** XRD patterns of final perovskite films without and with F-PMAI. **e** ToF–SIMS depth profiles show the distribution of respective elements over depth in the samples successively prepared by ITO/Spiro-TTB/perovskite with F-PMAI/C_60_/ALD SnO_2_, each layer corresponds to the feature element In (ITO), Br (perovskite), F (F-PMAI), C (Spiro-TTB, C_60_), Sn (SnO_2_), respectively

We added F-PMAI to the organic salt solution to prepare the perovskite solar cell, as shown in Fig. S1. We deduce that the molecular can interact with the uncoordinated lead and form hydrogen bonds with the organic cations [29, 30]. To verify the above-mentioned interactions, we first performed XPS to study the surface chemistry state of perovskite films (Fig. S3a). A clear F 1*s* peak at 687.5 eV, which belongs to F-PMA^+^, is observed in the doped film whereas it was absent in the control film in the XPS spectra (Fig. S3b), suggesting the successful introduction of F-PMAI into the perovskite. The Pb 4*f* peaks move toward lower binding energy after the addition of F-PMAI (Fig. 1a), indicating the intermolecular interaction between F-PMAI and PbI_2_. This may be from the interaction between electronegative F^−^ and uncoordinated Pb^2+^ [29]. We further analyzed the XPS spectra of I 3*d* and also found a lower binding energy shift (Fig. S3c). Such a shift is caused by the bonding between NH_3_^+^ from F-PMAI and I^−^ from [PbI_6_]^4−^ octahedra [31, 32]. Besides, we observed that the C=O peak (284.8 eV) originating from external oxygen/moisture was significantly suppressed after F-PMAI addition (Fig. S3d), and the hydrophobicity of perovskite film is also enhanced (Fig. S4), suggesting that the hydrophobic benzene ring with F ending groups structure is benefit to mitigating the degradation of perovskite [33]. To further investigate the interaction between F-PMAI and organic species in perovskites, we performed FTIR spectrometry of F-PMAI, FAI and F-PMAI/FAI mixture, respectively. As shown in Fig. 1b, the N–H stretching peak in F-PMAI and FAI is 3322 and 3325 cm^−1^, respectively, indicating almost the same position. However, it significantly shifted from 3325 to 3334 cm^−1^ for F-PMAI/FAI, and the C=N character stretching peak in FAI also shifted slightly from 1723 to 1722 cm^−1^ on adding F-PMAI, suggesting that the F-PMAI molecules form hydrogen bonds with organic cation FA^+^. The hydrogen bond consists of an electrostatic interaction between the hydrogen from organic salt and electronegative F (N–H···F) [30], which can effectively stabilize organic cations and reduce the generation of A-site vacancies.

SEM and XRD were performed to reveal how F-PMAI affects perovskite film quality. The PbI_2_ and CsCl were co-evaporated to form inorganic precursor films (Fig. S5a, b) and exhibit lead iodide (PbI_2_)-dominated crystalline structure, as confirmed by the XRD peak near 12.5° (Fig. S5c). Before forming the final perovskite films, three stages including organic salts deposition, pre-annealing and air-annealing were gone through. As illustrated in Fig. 1c, the air-annealing final perovskite film grains with F-PMAI were significantly enlarged than the control film. We also found perovskite films without and with F-PMAI exhibit different morphologies at organic salts deposition and pre-annealing stages as shown in Fig. S6. The control film shows a compact and randomly distorted morphology after drop-coating organic salts. Subsequently, the film further crystallized during the pre-annealing process and surface morphology has hardly changed (Fig. S6a), while for the additive film shows porous morphology after the addition of organic salts, and crystals began to precipitate in Fig. S6b. The difference in morphology demonstrated the much slower phase conversion for additive film, and the time of color change from yellow to black gave another evidence (Fig. S7). Notably, the peak area ratios of (001) PbI_2_/(100) perovskite for the additive film are larger than the control film at the stage of organic salt deposition and pre-annealing as shown in Fig. S8, indicating that a slower crystalline process for the additive film. This delayed nucleation kinetics can be attributed to the hydrogen bond interaction between F^−^ and FA^+^, thereby slowing down the reaction of PbI_2_ and FAI. Moreover, we found the peak positions of additive perovskite films are similar to those of control films, indicating that the F-PMAI does not enter the lattice and change the lattice parameters of the perovskite (Fig. 1d). There are no other diffraction peaks at low angles (2θ < 10°). Therefore, the addition of F-PMAI does not lead to the formation of 2D perovskite phase. The absence of 2D perovskite is beneficial for charge carrier transport and extraction throughout the perovskite absorber. ToF–SIMS were performed to observe the distribution of F-PMAI molecules in perovskite films, based on the structure ITO/Spiro-TTB/perovskite (F-PMAI)/C_60_/ALD SnO_2_ (Fig. 1e). Interestingly, a rising peak of F signal at the perovskite and Spiro-TTB interface is observed. This result suggests that the F-PMAI molecules are mostly extruded to the buried interface. This is related to the stronger Lewis acid–base interactions between the F-PMAI and Spiro-TTB. In short, the F-PMAI with large bulk cation cannot incorporate into the lattice, and be repelled to bottom and top interface, severing as a defect passivator via passivating undercoordinated Pb^2+^/I^−^ and A site vacancies defects. Hence, the schematic diagram of the F-PMAI interaction mechanism is shown in Fig. S9. F-PMA^+^ passivates uncoordinated Pb^2+^/I^−^ on the perovskite, inhibiting the production of deep-level defects and reducing the non-radiative recombination centers. F^−^ interacts with organic cations to form hydrogen bonds. In addition, I ions in F-PMAI also possibly fill the iodine vacancies [34]. Above, we propose that the F-PMAI retards crystallization by hydrogen bond interaction, and the larger steric hindrance of F-PMAI hinders the immediate reaction between the organic salt (e.g., FAI) and the precursor film (e.g., PbI_2_), improving the grain size. In terms of additive film, a higher critical Gibbs free energy (ΔG_c_) to nucleate contributes to fewer crystal nuclei and growth in more room into larger grains. This agrees with the appearance of a porous morphology at the film initial growth stage (Fig. S6b). The slow crystallization in additive film can provide a longer diffusion distance and self-assembly time between precursor salt ions and organic molecules, conducive to the formation of more complete reactions and larger grains as shown in Fig. S10.

### Orientation Regulation and Crystallization Mechanism of Perovskite Films

**Fig. 2:**
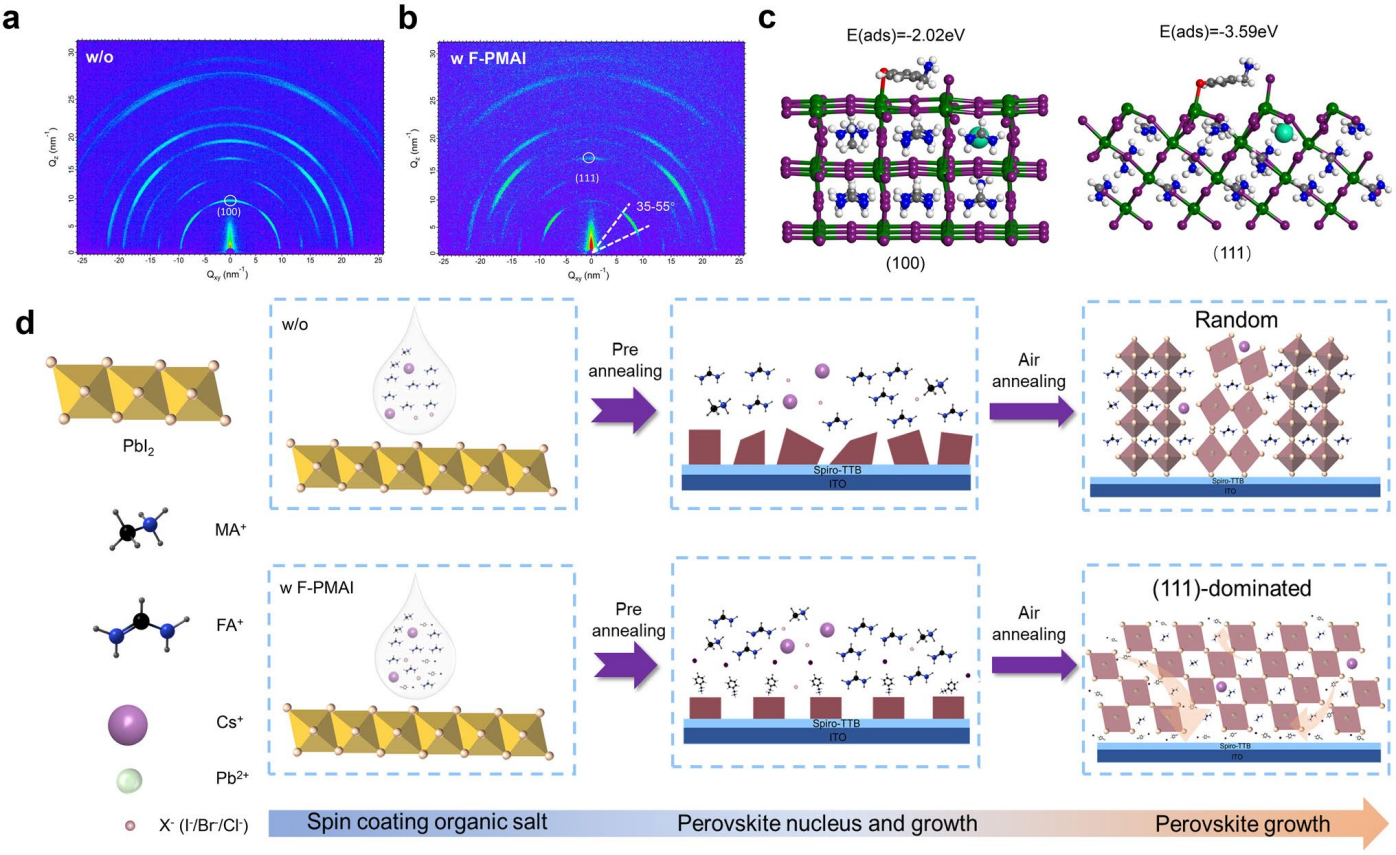
Crystallization and orientational preference studies of perovskite film with F-PMAI. 2D GIWAXS patterns of the perovskite films **a** without and **b** with F-PMAI. **c** Calculated models of F-PMAI adsorbed on (100) and (111) facets of perovskite and the corresponding adsorption energies. Green: Pb, purple: I, black: C, blue: N, white: H, red: F, cyan: Cs. **d** Schematic illustration of the perovskite crystal growth process without and with F-PMAI additive

In Fig. 1d, we notice an obvious (111) facet preferred orientation exhibited in the perovskite film with F-PMAI. The intensity ratio of (111)/(100) perovskite in the XRD pattern has been calculated as shown in Fig. S11. With the increase of F-PMAI concentration, the preference degree of (111) orientation also increases (Fig. S12). To further investigate the effect of the F-PMAI dopant on orientational preference of perovskite film, we performed GIWAXS measurements. The 2D diffraction patterns are shown in Fig. 2a, b. For the control perovskite film, the (100) plane showed the out-of-plane direction and other strong orientations, indicating irregular crystal orientation, in agreement with the above XRD and SEM discussed results. In contrast, the preferred orientation of (100) planes in the additive perovskite films changes from the out-of-plane (~ 90°) to ~ 45°-oriented direction, according to the integrated GIWAXS intensity plot azimuthally along the ring at a *q* value approximate to 10 nm^−1^ (Fig. S13). Simultaneously, the (111) planes preferentially orientate in the out-of-plane direction (Fig. 2b) [35]. This may be related to the interaction between F-PMAI and perovskite, resulting in a substantial crystal facet rotation along (111) out-of-plane directions [36]. We further conducted density functional theory (DFT) calculations to calculate the adsorption energy (*E*_ads_) of F-PMAI molecules on the (100) and (111) facets of perovskite, respectively. Shown in Fig. 2c, the calculated results indicate that the F-PMAI molecules have higher interaction energy with (111) than (100) facets (|E_ads_, _(111)_| >|E_ads_, _(100)_|), suggesting that the additive molecules are preferred to be adsorbed on (111) facets. The preferred adsorption of F-PMAI molecules can reduce the surface energy and facilitate the crystal growth of the (111) facet, leading to the perovskite film with exposed (111) facets [27].

Figure 2d shows the schematic illustration of the perovskite crystal growth process. After spin-coating organic salts on the inorganic precursor film, compared with the control, the film with F-PMAI initially forms fewer nuclei. In the subsequent pre-annealing stage, due to the presence of the bulky F-PMAI and its hydrogen bond interaction with the FA^+^, the organic salts react slowly with the inorganic salts in the precursor film, forming large-sized perovskite grains and preferred adsorption on the (111) facet to form (111) facet dominated perovskite crystal, which leads to grains stacking along the out-of-plane direction. During the subsequent air thermal annealing, the hydrogen bonds were broken, and the large F-PMAI molecules were repelled to the bottom and little to the top surface and the grain boundaries, severing as defects passivator. In this case, F-PMAI changes the nucleation and growth pathways to give a strong growth orientation perpendicular to the substrate surface, as shown in the cross-sectional SEM images of Fig. S10b, which promises to improve the film crystal quality, reduce film defects, facilitate the transport of charge carriers and achieve higher device performance.

### Defect Passivation and Charge Carrier Dynamics

**Fig. 3:**
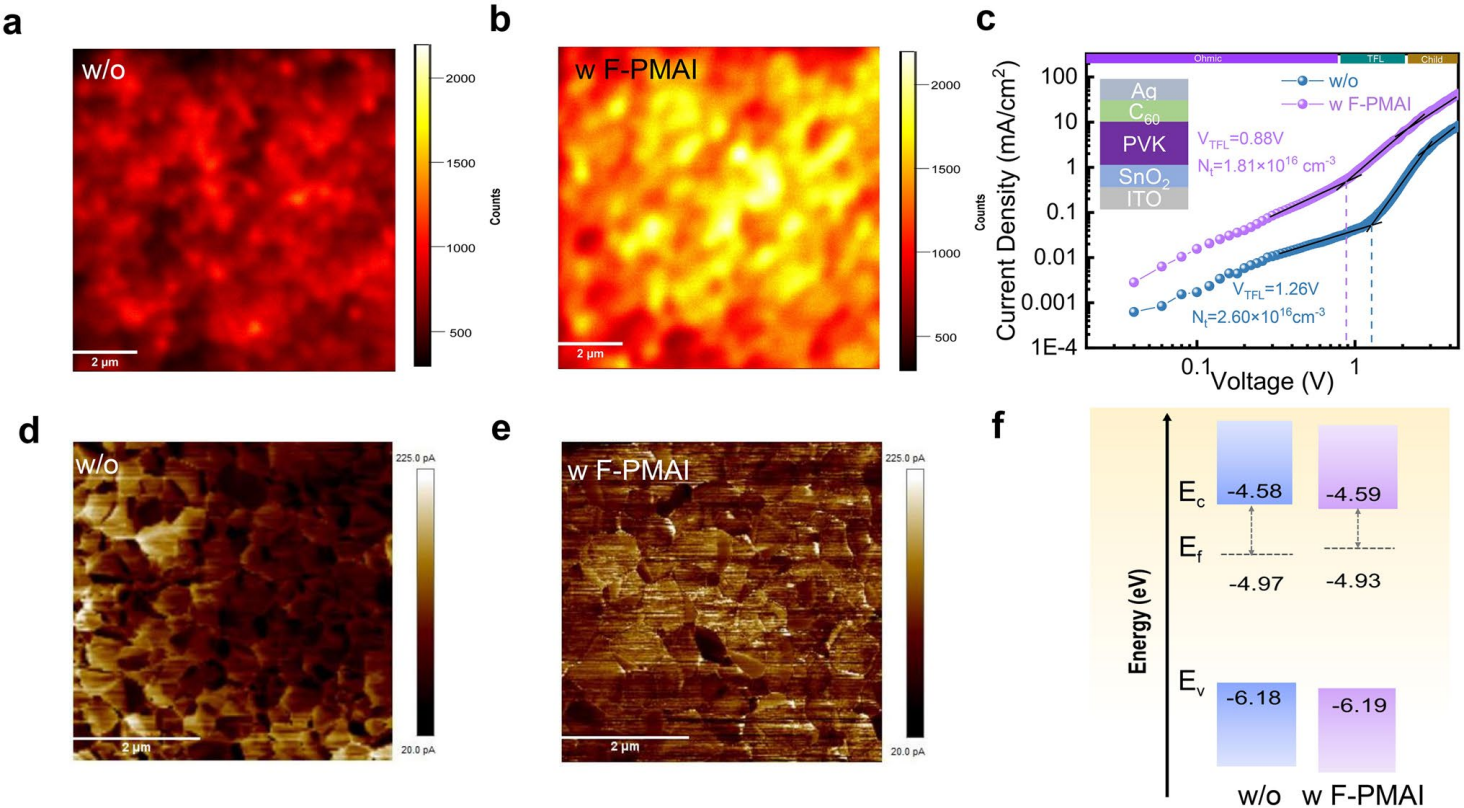
The effect of F-PMAI on defect and carrier dynamic. PL mapping of the perovskite films **a** without and **b** with F-PMAI with a structure of ITO/perovskite. **c** SCLC curves of devices without and with F-PMAI. C-AFM images of perovskite films **d** without and **e** with F-PMAI. **f** Energy-level scheme of perovskite films without and with F-PMAI based on the parameters derived from UPS spectra

We measured the PL and TRPL to explore how F-PMAI affects perovskite film quality. Perovskite film with F-PMAI exhibits stronger PL intensity and longer carrier lifetime from 252.20 to 536.38 ns than control film (Fig. S14a, b and Table S1), indicating better crystal quality and suppression of non-radiative charge recombination in film with F-PMAI. Furthermore, the PL mapping test was used to better understand the effect of F-PMAI on charge carrier extraction and transport dynamics. Perovskite film with F-PMAI deposited on ITO substrate exhibited a higher PL intensity than control film (Fig. 3a, b). The results show that F-PMAI significantly decreased defect density and suppressed trap-assisted non-radiative recombination. This is further confirmed by the densities of trap states (N_t_) of perovskite film using SCLC measurement, with the structure of ITO/SnO_2_/perovskite with or without F-PMAI/C_60_/Ag for electron-only device, as shown in Fig. 3c. Compared to the *V*_TFL_ value of the control film (1.26 V), the film with F-PMAI has a smaller* V*_TFL_ value of 0.88 V. The *N*_t_ of samples without and with F-PMAI is 2.60 × 10^16^ and 1.81 × 10^16^ cm^−3^, respectively [32]. The lower trap density can be attributed to the decreased grain boundaries and associated defects by enlarged grain size and the reduced undercoordinated Pb^2+^/I^−^ defects via passivation of F-PMAI. Considering that F-PMAI is distributed at both bottom and top of the perovskite film, we also investigated the charge extraction between perovskite film and charge transport layer. Perovskite film with F-PMAI deposited on ITO/Spiro-TTB (hole transport layer (HTL)) substrate and ITO/perovskite with F-PMAI/C_60_ (electron transport layer (ETL)) both exhibit a PL quenching than control film, indicating enhanced charge transfer from perovskite to HTL/ETL, respectively (Fig. S15) [36, 37]. It is expected to improve interfacial contact and effectively passivate interface defects. Moreover, the impact of F-PMAI on charge carrier dynamics was investigated using C-AFM. Compared to the control film, the dark current is significantly and uniformly enhanced in the perovskite film with F-PMAI, with the average current value increasing from 96.4 to 113 pA (Fig. 3d, e). This enhancement indicates more efficient charge transport, which can be attributed to the superior electrical properties of (111) facets as reported previously [38]. The KPFM was measured to evaluate the distribution of the surface potential of perovskite films (Fig. S16a, b), which shows a decrease of work function (WF) in the perovskite film with F-PMAI, indicating a more n-type nature. This change matches well with the results obtained from UPS, showing the Fermi level (*E*_F_) closer to the conductive band edge (Fig. S17). Figure 3f shows the energy-level scheme of perovskite films without and with F-PMAI. The more n-characteristic film surface stems from the improved crystal quality and modified surface termination after adding F-PMAI, enabling more efficient electron extraction between the perovskite and ETL [39, 40].

### Single Junction Device Performance

**Fig. 4:**
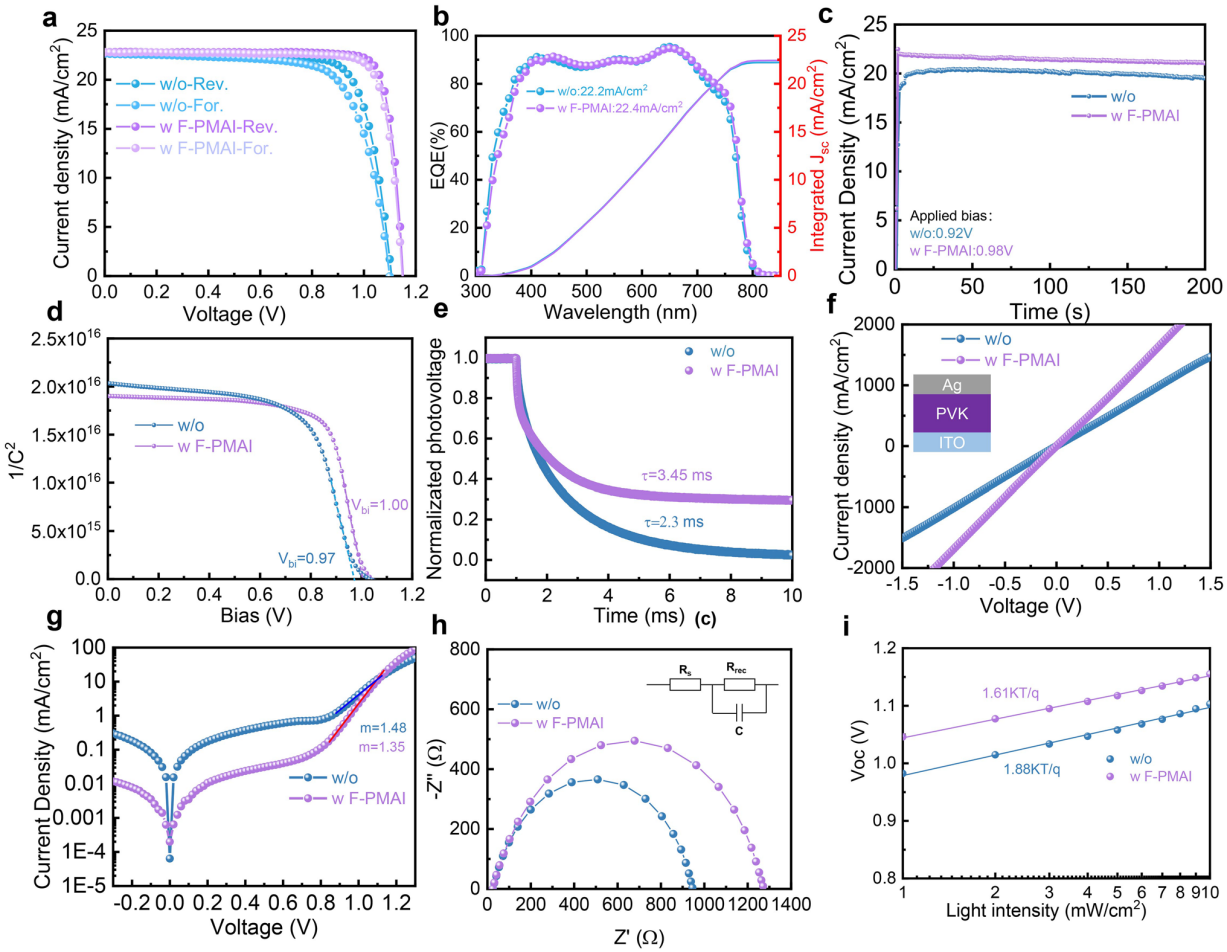
The influence of F-PMAI on PSCs performance. **a** *J–V* curves of the champion devices without and with F-PMAI. Rev.: reverse scan; For.: forward scan. **b** EQE curves of devices without and with F-PMAI. **c** Stabilized power output of PSCs without and with F-PMAI. **d** Mott–Schottky plots, **e** TPV decay curves of PSCs without and with F-PMAI. **f** Vertical *J–V* curves of ITO/perovskite without and with F-PMAI/Ag. **g** Dark *J–V* curves, **h** Nyquist curves, **i** dependence of *V*_OC_ on light intensity of PSCs without and with F-PMAI

We fabricated CsFAMAPbI_3−x_Br_x_ PSCs based on inverted p–i–n architecture ITO/Spiro-TTB/perovskite/C_60_/BCP/Ag to evaluate the effects of the additive on device performance. The preparation process of perovskite solar cells shown in Fig. S1 and the other layers are prepared by a thermal evaporation process. The optimized concentration of F-PMAI is 1.5 mol% (Fig. S18). Increasing the concentration of F-PMAI enhances the (111) orientation, but it can also cause a large number of F-PMAI to hinder sufficient reaction and accumulate in the interface, leading to the precipitation of PbI_2_ as shown in Fig. S12. These features ultimately lead to a decrease in device performance, the PV parameters of perovskite solar cells are shown in Fig. S18. Encouragingly, F-PMAI substantially improved the open-circuit voltage (*V*_OC_) and fill factor (*FF*) of devices. A champion device using F-PMAI exhibits *V*_OC_ up to 1.15 V, a gain of 50 mV compared with the control device in Fig. 4a and Table 1. Accompanied by a noticeable *FF* improvement (77.43% to 85.48%), the champion F-PMAI device achieved a high PCE of 22.74%. The integrated current density with F-PMAI and without F-PMAI from the EQE is 22.4 and 22.2 mA cm^−2^ (Fig. 4b), respectively, which well match with the *J*_sc_ extracted from *J–V* curves. The hysteresis index (HI) is defined as ((PCE_reverse_– PCE_forward_)/PCE_reverse_ × 100%), decreasing from 7% in the control to 2% in the additive device. The stabilized power output (SPO) of the F-PMAI device is 21.2% at the maximum power point (MPP) based on a *V*_max_ of 0.98 V (Fig. 4c). We investigated the light stability of PSCs with F-PMAI using an LED solar simulator with an intensity equivalent to 1-sun illumination at 25 °C in ambient conditions. The unencapsulated F-PMAI device maintained 95% of initial PCE after 600 h under continuous light soaking (Fig. S19). This reduced hysteresis and increased photostability may benefit from reduced defects and suppressed ion migration, so we calculated the ion migration activation energy (*E*_a_) using temperature-dependent conductivity (Fig. S20). The additive perovskite film is found to show a higher *E*_a_ of 262 meV compared with the control (199 meV). This indicates that ions are harder to migrate in the perovskite layer with F-PMAI than in the control [27, 36, 38, 41].

**Table 1:** PV parameters of PSCs without and with F-PMAI

Sample	Scan direction	*V*_oc_ (V)	*J*_sc_ (mA cm^−2^)	*FF* (%)	PCE (%)	SPO (%)	HI (%)
w/o	Rev.	1.10	22.65	77.43	19.38	18.6	7
	For.	1.09	22.66	72.15	17.95		
**w F-PMAI**	**Rev.**	**1.15**	**22.77**	**85.48**	**22.74**	**21.2**	**2**
	**For.**	**1.14**	**22.82**	**83.00**	**22.07**		

To further investigate the reason for the improvement in device efficiency, we systematically studied the charge carrier behavior and defect physics of devices without and with F-PMAI. First, we performed capacitance–voltage (*C–V*) measurements to directly compare the built-in potential (*V*_bi_) of devices. The control device has a *V*_bi_ of 0.97 V, while the additive device has a higher *V*_bi_ of 1.00 V, in agreement with the *V*_oc_ enhancement shown in Fig. 4d. The larger the *V*_bi_ is, the more favorable for carrier separation and transfer, which reduces the charge accumulation at the perovskite/C_60_ interface, reducing the hysteresis [32]. We attribute the increased potential to defect passivation and improved energy level at the perovskite/C_60_ interface. The TPV measurement result in Fig. 4e shows that the carrier lifetime significantly increases from 2.3 to 3.45 ms after adding the F-PMAI, which further proves the suppressed carrier non-radiative recombination [42, 43]. To identify the effect of F-PMAI additive on *FF* of the device, we investigated the electrical conductivity of perovskite film without and with F-PMAI by vertical dark *J–V* measurements with a structure of ITO/perovskite/Ag (Fig. 4f). Adding the F-PMAI additive resulted in higher electrical conductivity and yielded a higher *FF* [44]. We calculated the ideally factor (m) of the device in the dark *J–V* curves (Fig. 4g). The value reduces from 1.48 in the control to 1.35 in the device with F-PMAI. This suggests suppressed non-radiative recombination [45], which is consistent with the increased recombination resistance (*R*_rec_) in the EIS (Fig. 4h). It is well known that the *R*_rec_ values can reflect the degree of charge recombination in a device, which is typically correlated to the defect state density of the perovskite film. In general, a larger *R*_rec_ signifies a more difficult charge carrier recombination and results in a higher *V*_OC_ [46]. To further elucidate the charge carrier recombination dynamics of PSCs, we conducted light intensity-dependent *V*_OC_ measurement (Fig. 4i). Compared with the slope of 1.88 *KT/q* for the control device, the slope decreases to 1.61 *KT/q* for the additive device, revealing that the defect-assisted recombination is effectively suppressed due to reduced trap density [28]. The above calculation methods can be found in Supporting Information. The above results show that F-PMAI improves the perovskite film crystalline quality, passivates interface defects, effectively suppresses non-radiative recombination, and enhances carrier transport properties of devices, which leads to improved *J*_SC_, *V*_OC_ and *FF*.

### Tandem Device Performance and Stability

**Fig. 5:**
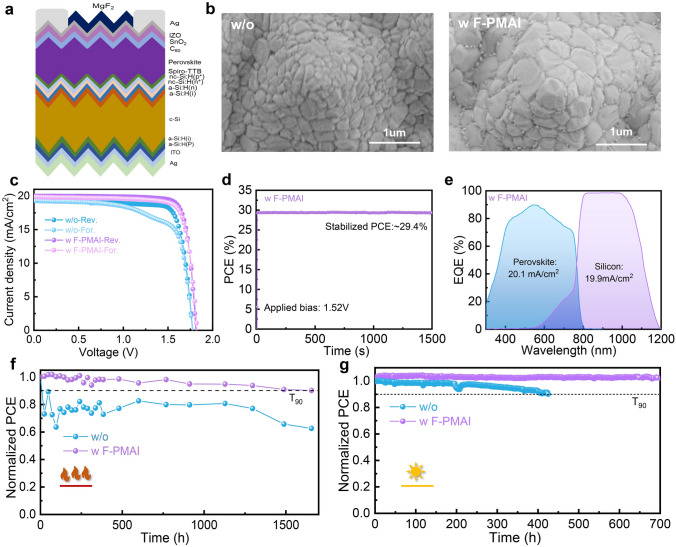
The effect of F-PMAI on TSCs performance and stability. **a** Schematic structure of monolithic perovskite/silicon tandem solar cells. **b** Top-view SEM of monolithic perovskite/silicon tandem solar cells without and with F-PMAI. **c** *J–V* curves of the champion devices without and with F-PMAI. **d** Stabilized power output and **e** EQE curves of TSCs with F-PMAI. **f** Thermal stability of TSCs without and with F-PMAI under 85 °C N_2_ glove box. T_90_ represents the lifetime at which the PCE declines to 90% of its initial value. **g** Light stability of TSCs without and with F-PMAI under ambient conditions (30%–40% relative humidity (RH), ~ 25 °C). The data are normalized to their initial efficiency

Inspired by the substantial enhancement in single-junction devices using F-PMAI, we used our additive perovskite films to fabricate monolithic perovskite/silicon TSCs. The Si bottom cells are industrially feasible double-side fully textured silicon solar cells. The detailed fabrication process is described in the experimental section, and the schematic diagram of the tandem structure is illustrated in Fig. 5a. The enlarged grain size can also be found in the tandem devices with F-PMAI (Figs. 5b and S21). Energy-dispersive X-ray spectroscopy (EDS) mapping shows that F-PMAI molecules are distributed on the bottom and top of perovskite films, suggesting similar crystal and passivation effects can also be produced on textured silicon bottom cells (Fig. S22). Therefore, this additive can also be explored for improving TSCs efficiency as shown in Fig. S23, F-PMAI significantly enhances the * V*_OC_ and *FF* of TSCs as well as improves PCE. Encouragingly, TSC with F-PMAI reaches a reverse-scan PCE of 30.05% with a *V*_OC_ of 1.81 V, a *J*_SC_ of 20.01 mA cm^−2^, and an *FF* of 82.91% as shown in Fig. 5c and Table 2. The efficiency represents the best result reported to date for industrially feasible textured silicon tandem solar cells based on nanocrystalline silicon tunneling junction (nc-Si:H) (Table S2). The HI decreases from 13% in the control to 2% in the additive TSCs, attributed to the improved crystal quality and defect passivation in the perovskite films with F-PMAI. MPP tracking yields a steady-state PCE of 29.4% up to 1500 s (Fig. 5d). The EQE spectra show integrated* J*_SC_ of 20.1 and 19.9 mA cm^−2^ for top and bottom subcells, respectively, basically consistent with the *J–V* results (Fig. 5e).

**Table 2:** Champion PV parameters of TSCs without and with F-PMAI

Sample	Scan direction	*V*_oc_ (V)	*J*_sc_ (mA cm^−2^)	*FF* (%)	PCE (%)	HI (%)
w/o	Rev.	1.77	19.32	80.11	27.61	13
	For.	1.77	19.33	69.69	24.01	
w F-PMAI	Rev.	1.81	20.01	82.91	30.05	2
	For.	1.82	19.91	80.63	29.32	

Long-term stability is important to the industrialization of perovskite photovoltaics. Therefore, we investigated the effects of F-PMAI on the long-term stability of TSCs. The thermal stability of unencapsulated tandem devices was performed on a hot plate at 85 °C in a dark nitrogen-filled glovebox. The control device reduced to less than 80% of initial efficiency after 24 h and fluctuated, whereas that of the F-PMAI device showed a very small PCE decay and retained 90% of initial PCE after 1650 h, indicating improved thermal stability (Fig. 5f). We further tracked the long-term light stability of tandem devices in Fig. 5g, which shows that the PCE of the unencapsulated devices with F-PMAI still retained its initial PCE after 700 h tracking in ambient conditions (RH: 30%–40%, 25 °C). The enhanced stability of TSCs with F-PMAI is attributed to better perovskite film quality and all-around defect passivation. If more efficient encapsulation techniques and optimal perovskite compositions are used, further improved efficiency and stability of TSCs can be expected to meet the requirements for practical applications.

## Conclusions

In summary, we explored a multi-functional strategy for achieving high-quality and stable perovskite films by employing a large organic molecule additive, F-PMAI, which can form hydrogen bond to retard the perovskite crystallization process and preferably adsorb on (111) facets to reduce the surface energy. The resulting perovskite films have large grain size and (111) preferred crystal orientation. Subsequently, the F-PMAI molecules are mainly repelled to the bottom and top surface of the perovskite films after growth, function as an efficient interface passivation and promote carrier transport at the two interfaces. Finally, using this high-quality perovskite film on full-textured silicon substrates with nc-Si:H tunneling junctions, we fabricated champion efficiency of 30.05% perovskite/silicon TSC. Moreover, the stability of perovskite is greatly improved. Unencapsulated TSCs show excellent thermal stability retaining 90% initial PCE after 1650 h under the N_2_ glovebox and superior light stability maintaining its initial PCE after 700 h. This strategy provides valuable guidance for selecting suitable additives to further realize efficient and stable TSCs toward final practical deployment.

## Supplementary Information

Below is the link to the electronic supplementary material.

Supplementary file1 (DOCX 3366 kb)

Supplementary file2 (PDF 4025 kb)
