# Supplementary material for: Textured Perovskite/Silicon Tandem Solar Cells Achieving Over 30% Efficiency Promoted by 4-Fluorobenzylamine Hydroiodide
Source: Nanomicro Lett. 2024 May 2;16:189. doi: 10.1007/s40820-024-01406-4 (PMC11065830; doi:10.1007/s40820-024-01406-4)
Supplement: Supplementary file 1 — Supplementary file1 (DOCX 3366 kb) [file 40820_2024_1406_MOESM1_ESM.docx]

Supporting Information for

**Textured Perovskite/Silicon Tandem Solar Cells Achieving Over 30% Efficiency Promoted by 4-Fluorobenzylamine Hydroiodide**

Jingjing Liu^1,2,3,4,5^, Biao Shi^1,2,3,4,5,^*, Qiaojing Xu^1,2,3,4,5^, Yucheng Li^1,2,3,4,5^, Yuxiang Li^1,2,3,4,5^, Pengfei Liu^1,2,3,4,5^, Zetong SunLi^1,2,3,4,5^, Xuejiao Wang^1,2,3,4,5^, Cong Sun^1,2,3,4,5^, Wei Han^1,2,3,4,5^, Diannan Li^1,2,3,4,5^, Sanlong Wang^1,2,3,4,5^, Dekun Zhang^1,2,3,4,5^, Guangwu Li^6,7^, Xiaona Du^1,2,3,4,5^, Ying Zhao^1,2,3,4,5^, and Xiaodan Zhang^1,2,3,4,5,^ *

^1^ Institute of Photoelectronic Thin Film Devices and Technology, Renewable Energy Conversion and Storage Center, Solar Energy Conversion Center, Nankai University, Tianjin 300350, P. R. China

^2^ Key Laboratory of Photoelectronic Thin Film Devices and Technology of Tianjin, Tianjin 300350, P. R. China

^3^ Haihe Laboratory of Sustainable Chemical Transformations, Tianjin 300192, P. R. China

^4^ Engineering Research Center of Thin Film Photoelectronic Technology of Ministry of Education, Tianjin 300350, P. R. China

^5^ Collaborative Innovation Center of Chemical Science and Engineering (Tianjin), Tianjin 300072, P. R. China

^6^ Center of Single-Molecule Sciences, Institute of Modern Optics, Tianjin Key Laboratory of Micro-Scale Optical Information Science and Technology, College of Electronic Information and Optical Engineering, Nankai University, 38 Tongyan Road, Jinnan District, Tianjin 300350, P. R. China

^7^ Shenzhen Research Institute of Nankai University, 16th Floor, Yantian Science & Technology Building, Haishan Street, Yantian District, Shenzhen, 518083, P. R. China

*Corresponding authors. E-mail: biaos_xiaog@163.com (Biao Shi), xdzhang@nankai.edu.cn (Xiaodan Zhang)

**S1 Calculation Methods**

**S1.1 Simulation Calculation**

First-principle calculations were performed by density functional theory (DFT) using the Vienna Ab-initio Simulation Package (VASP) package [S1]. The generalized gradient approximation (GGA) with the Perdew-Burke-Ernzerhof (PBE) functional was used to describe the electronic exchange and correlation effects [S2–S4]. Uniform G-centered k-point meshes with a resolution of 2π×0.05 Å^-1^ and Methfessel-Paxton electronic smearing were adopted for the integration in the Brillouin zone for geometric optimization. The simulation was run with a cutoff energy of 500 eV throughout the computations. These settings ensure convergence of the total energies to within 1 meV per atom. Structure relaxation proceeded until all forces on atoms were less than 10 meV Å^-1^ and the total stress tensor was within 0.03 GPa of the target value. The DFT-D2 Van der Walls correction by Grimmie [S5, S6] was also considered in all calculations.

The adsorption energies of F-PMAI molecule adsorbed on FA/MA/Cs-PbI_3_ (100) and (111) surfaces were calculated by the following equation: ΔE_(ads)_=E_(total)_-E_(surface)_-E_(F-PMAI)_, where E_(total)_ is the energy of F-PMAI molecule adsorbed on FA/MA/Cs-PbI_3_ (100) and (111) surfaces, E_(surface)_ is the energy of FA/MA/Cs-PbI_3_ (100) and (111) surfaces, and E_(F-PMAI)_ is the energy of the F-PMAI molecule. The more negative the value, the stronger the binding ability.

**S1.2 Ion Migration Activation Energy (*E*_a_) Calculation**

Temperature-dependent conductivity, σ (T), was measured to compare the activation energy (E_a_), for ion migration. E_a_ can be calculated from Equation [S7, S8]$\sigma\left( T \right)T=\sigma_{o}e^{(-\frac{E_{a}}{KT})} ,$Where k is the Boltzmann’s constant, $\sigma_{o}$ is the constant, and T is the temperature. Based on ln($\sigma\left( T \right)T)$versus1000/T plots, the Ea for ion migration was extracted from the slope of the fitted lines at relatively higher temperature.

**S1.3 Space Charge Limited Current (SCLC) Measurements**

The trap densities were extracted using the equation: $N_{t}=\frac{2\varepsilon\varepsilon_{0}V_{TFL}}{eL^{2}}$, where $N_{t}$ denotes the trap state density, ε and $\varepsilon_{0}$ are the relative dielectric constant and the vacuum dielectric constant, respectively, $V_{TFL}$ is the trap-filled limit voltage, e is the electron charge and L is the thickness of perovskite film [S9].

**S1.4 Capacitance-voltage (C-V) Measurements**

The Mott-Schottky equation: $\frac{1}{C^{2}}=\frac{2\left( V_{bi}-V \right)}{A^{2}e\varepsilon\varepsilon_{0}N_{A}}$(A is the device area, $\varepsilon$ and$\varepsilon_{0}$ are the relative and vacuum permittivity, and $N_{A}$is carrier concentration) [S10].

**S1.5 Dark *J-V* Curves Measurement**

In the dark *J-V* curves, the ideal factor (m) is extracted from the equation: $m=(\frac{KT}{q}\frac{dlnJ}{dV})$. When m=1, bimolecular recombination dominates; when m=2, trap-assisted recombination dominates [S11].

**S1.6 Light Intensity-dependent *V*_oc_ Measurement**

The slope of the dependence of V_oc_ verse light intensity (I) is used to evaluate the degree of the trap-assisted recombination via the equation $V_{oc}=\frac{nKTln\left( I \right)}{q}+c$ (n is the ideal factor, K is the Boltzmann constant, T is the absolute temperature, I is the incident light intensity, q is the elementary charge, and c is the constant) from the Shockley−Read−Hall recombination mechanism. The closer the value of n to 1, the less trap-assisted nonradiative recombination exists in the PSCs [S12].

**S2 Supplementary Figures**


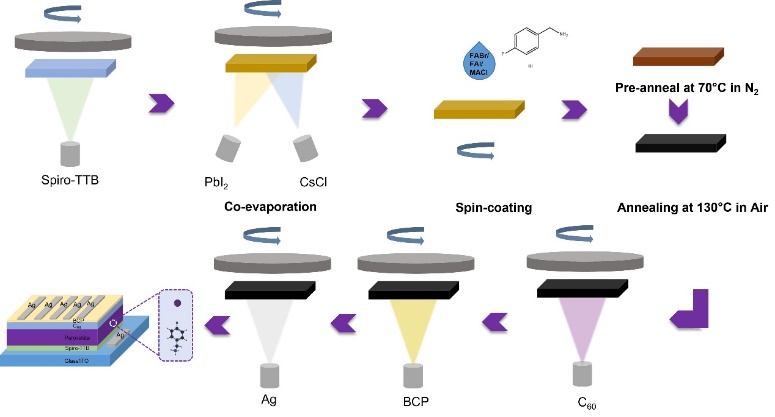


**Fig. S1** Schematic of the hybrid two-step deposition perovskite solar cells


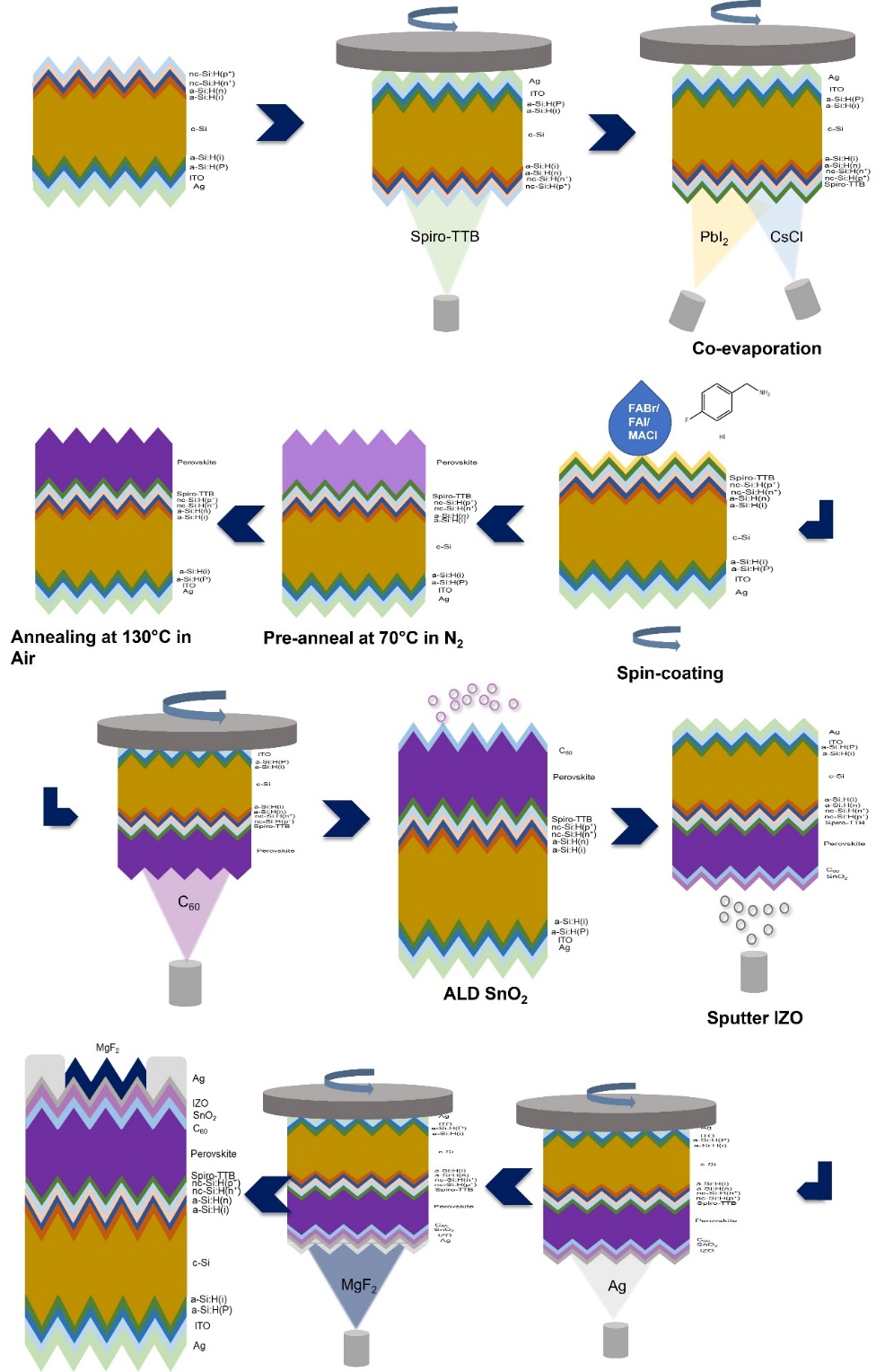


**Fig. S2** Schematic of the fabrication of perovskite/silicon tandem solar cells


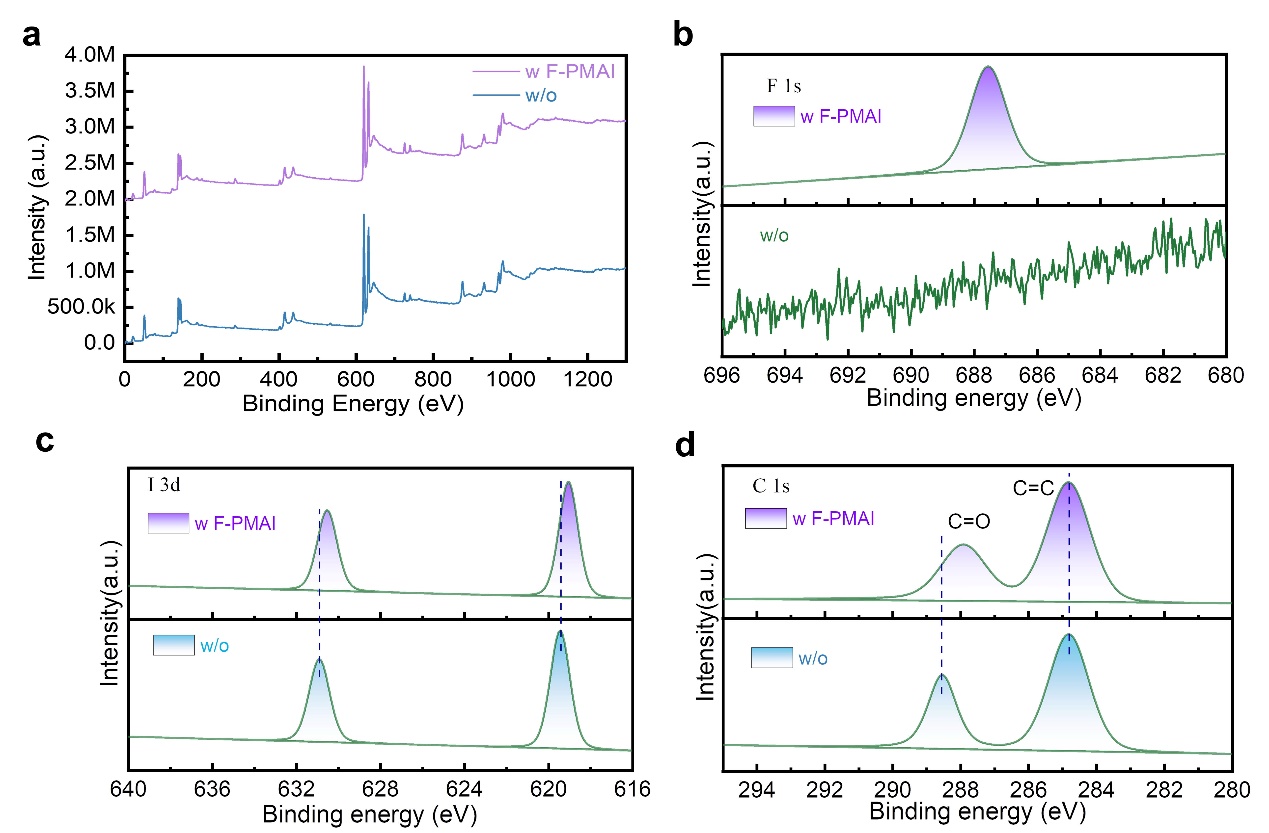


**Fig. S3** **a)** Full-scale X-ray photoelectron spectroscopy (XPS) spectrum of perovskite films without and with F-PMAI. XPS spectra of **b)** F 1s, **c)** I 3d and **d)** C 1s of perovskite films without and with F-PMAI additive


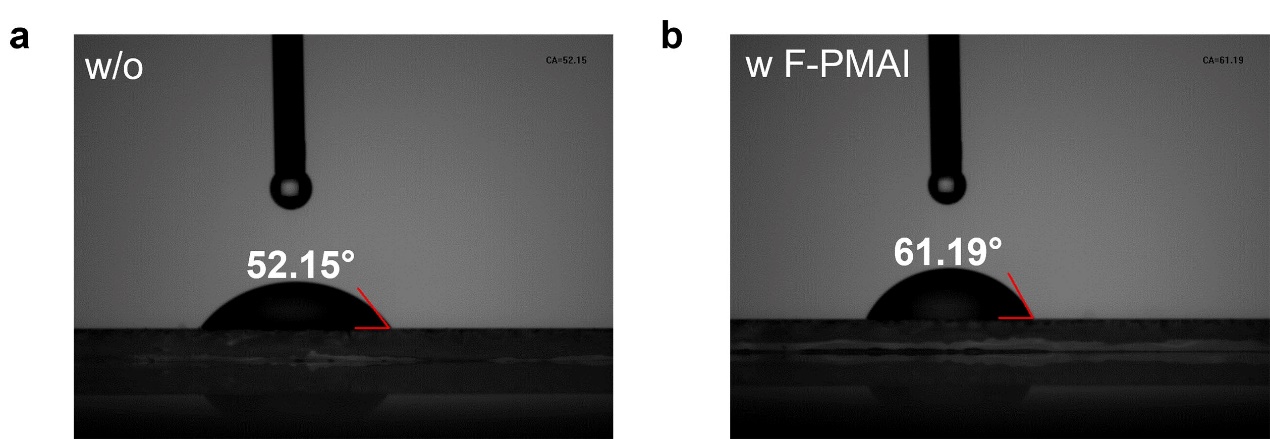


**Fig. S4** Images of water droplets on the surface of perovskite films **a)** without and **b)** with F-PMAI


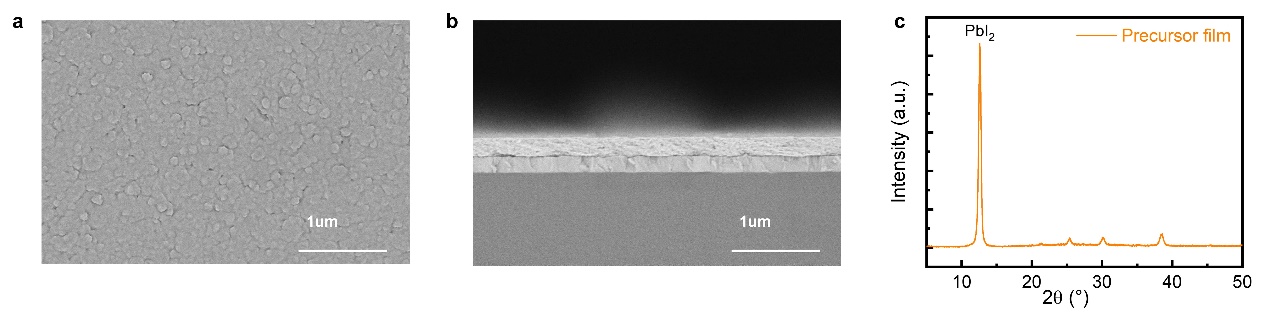


**Fig. S5** Top-view **a)** and cross-sectional **b)** SEM images of co-evaporated precursor films. **c)** XRD spectra of co-evaporated precursor films


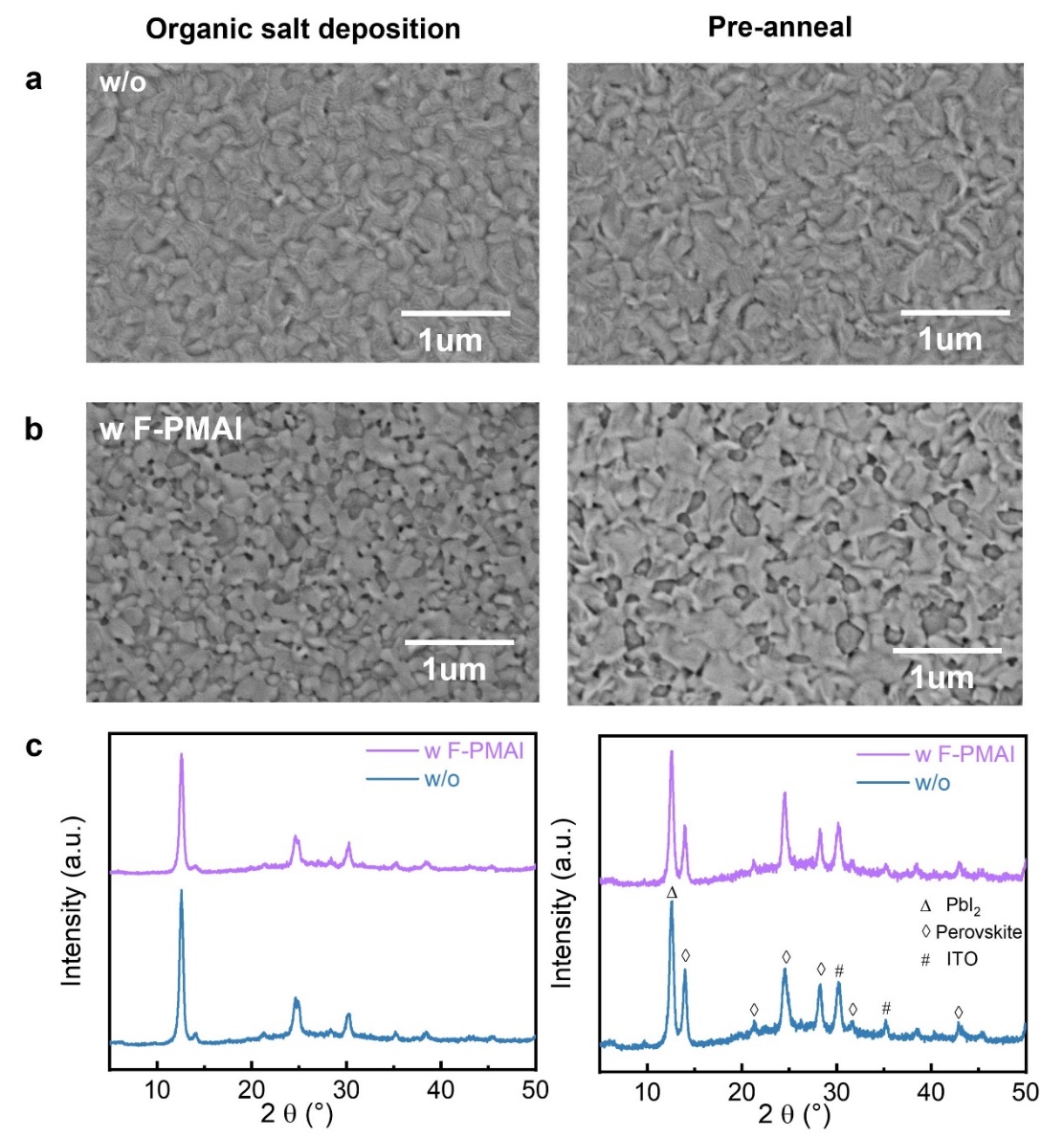


**Fig. S6** Top-view and SEM images of perovskite films **a)** without and **b)** with F-PMAI during the different processes. **c)** XRD patterns of perovskite films without and with F-PMAI after different processes, including organic salt deposition and pre-annealing


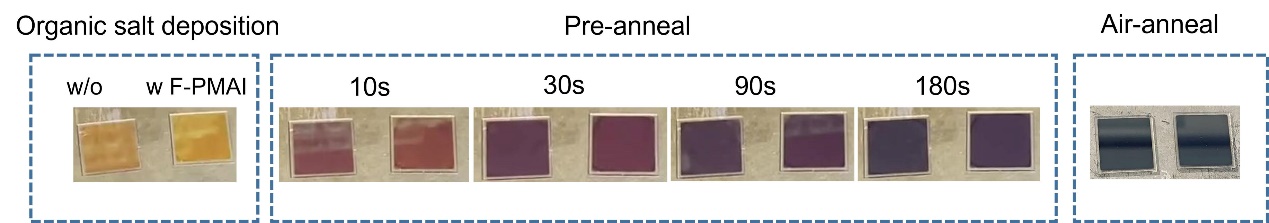


**Fig. S7** Photographs of perovskite thin films without and with additives at different processes and time


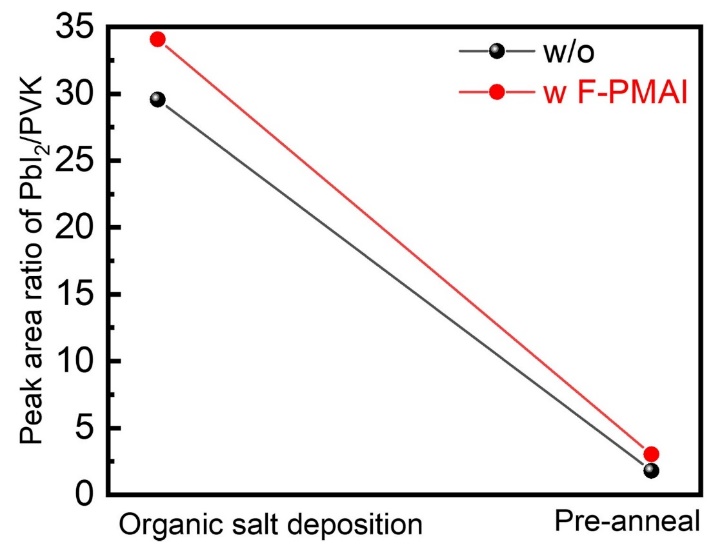


**Fig. S8** The peak intensity ratio of PbI_2_/PVK at the organic salt deposition and pre-anneal stage respectively


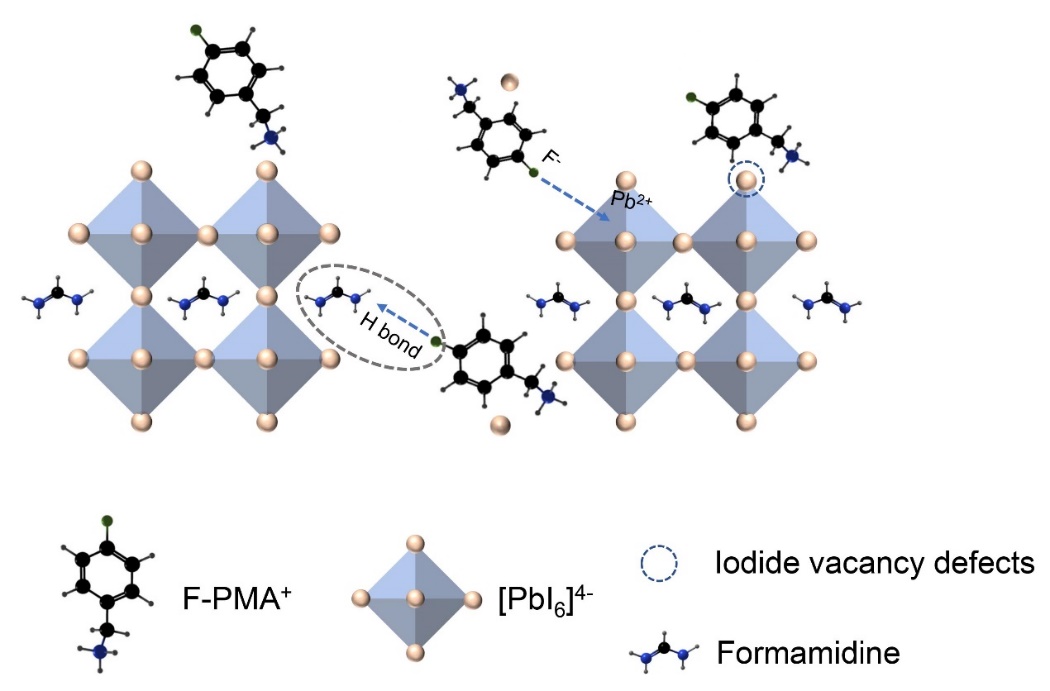


**Fig. S9** Schematic diagram of interaction mechanism of the F-PMAI on perovskite


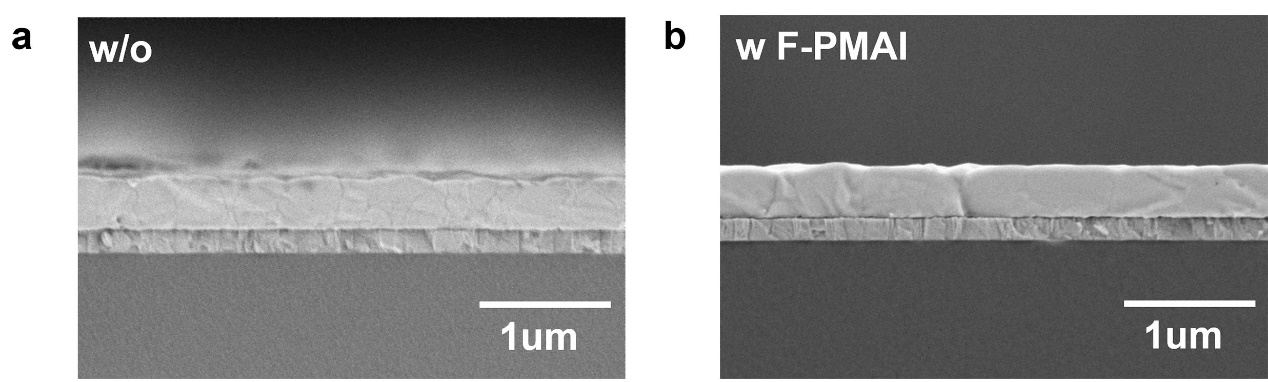


**Fig. S10** Cross-section SEM images of perovskite films **a)** without and **b)** with F-PMAI


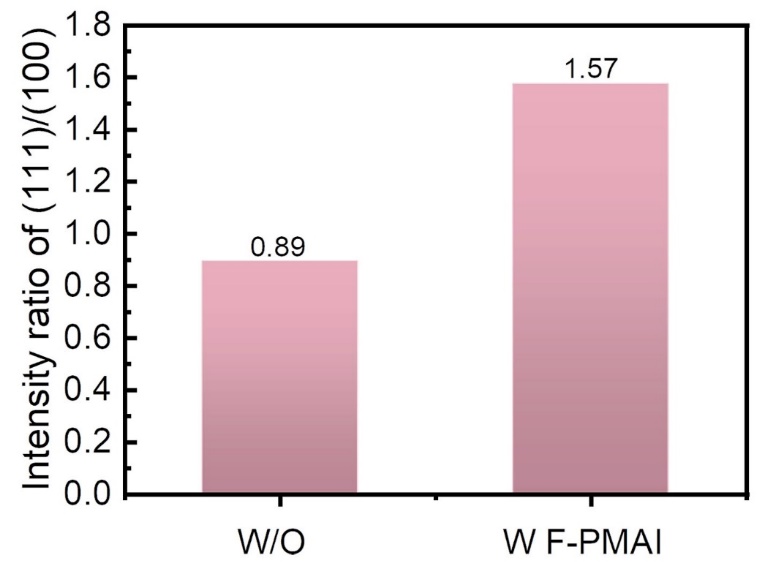


**Fig. S11** Peak intensity ratio of (111) and (100) perovskite in Fig. 1d


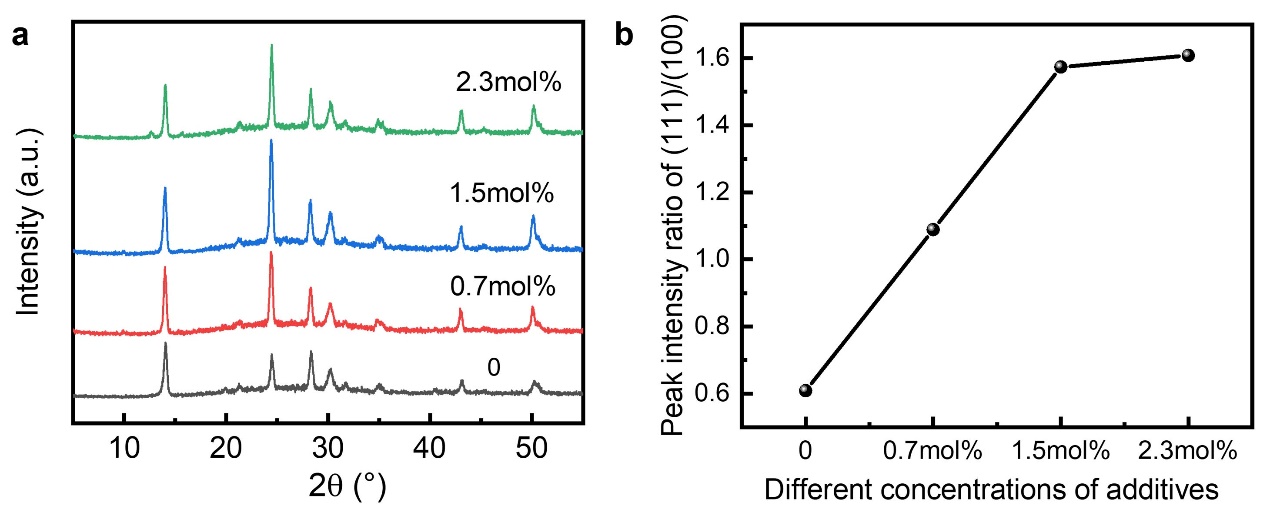


**Fig. S12** **a)** XRD pattern of perovskite film with different F-PMAI concentrations (0, 0.7mol%, 1.5mol%, 2.3mol%). **b)** Peak intensity ratio of (111) and (100) perovskite


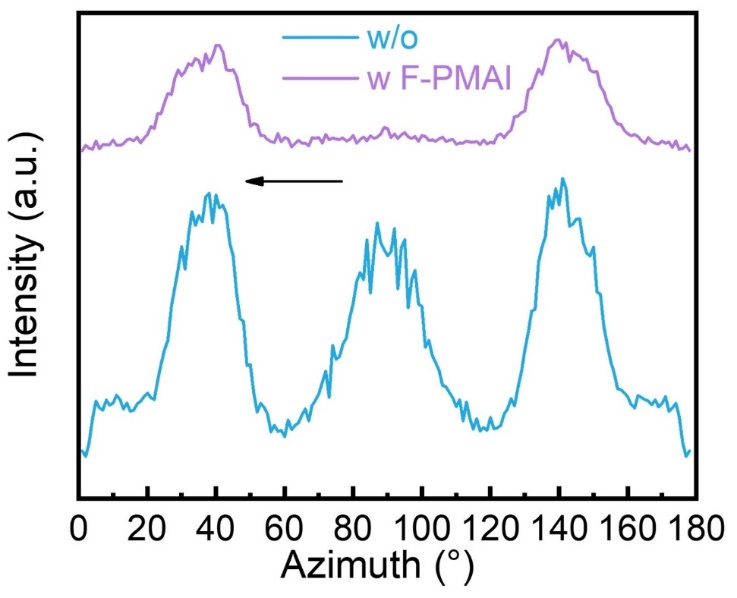


**Fig. S13** Integrated GIWAXS intensity plots azimuthally along the ring at a q≈10 nm^−1^, assigned to the (100) plane of perovskite films without and with F-PMAI


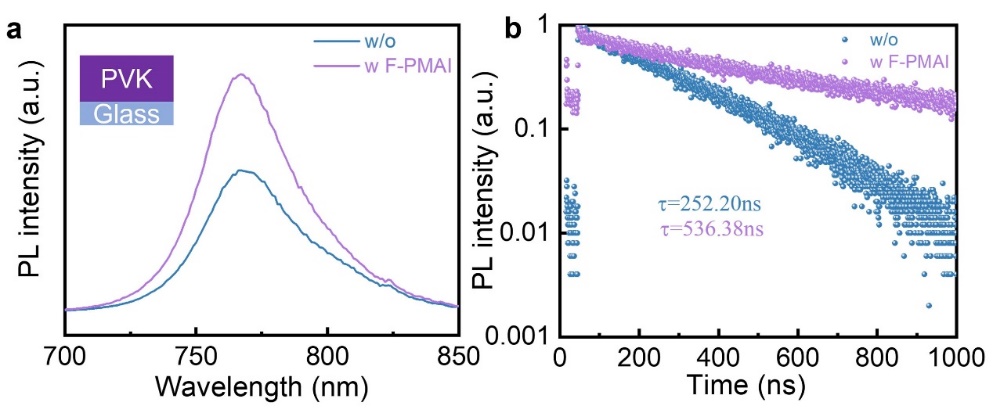


**Fig. S14** **a)** PL and **b)** TRPL spectra of perovskite films without and with F-PMAI deposited on ITO substrates


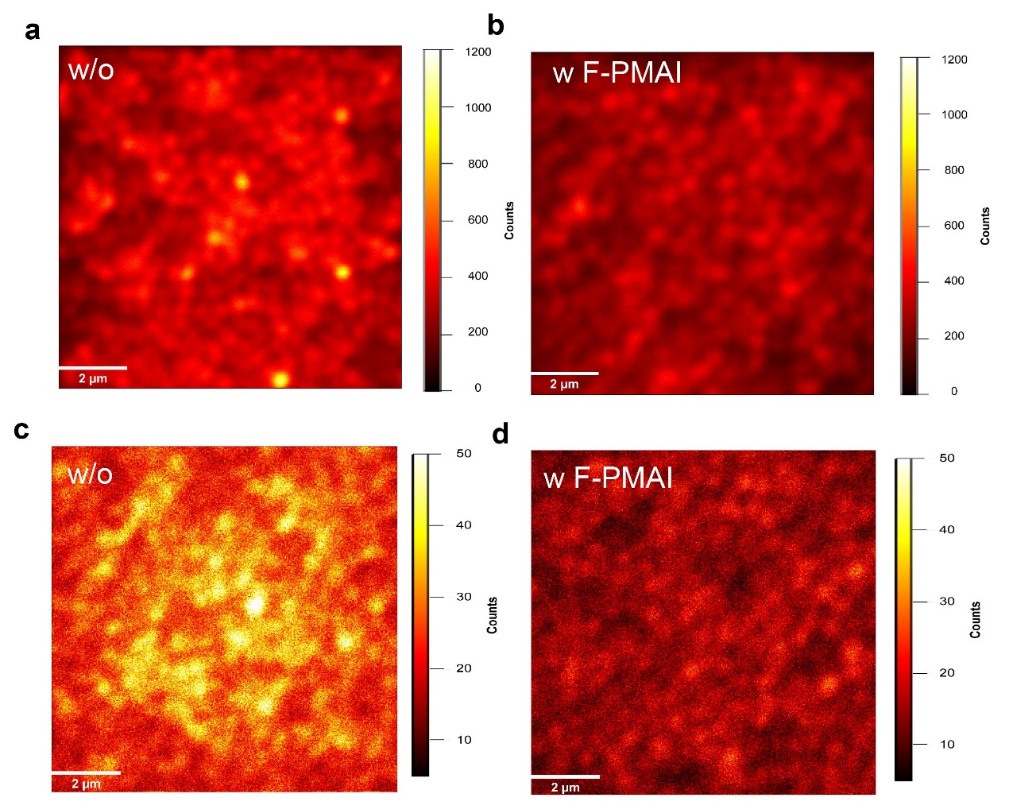


**Fig. S15** PL mapping of perovskite films **a)** without and **b)** with F-PMAI with a structure ITO /Spiro-TTB /perovskite. PL mapping of perovskite films **c)** without and **d)** with F-PMAI with a structure of ITO/perovskite/C_60_


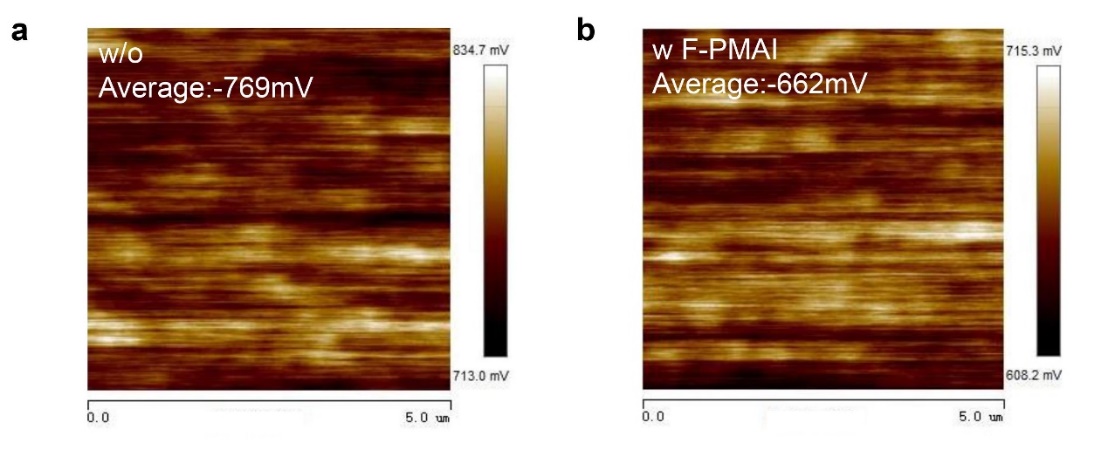


**Fig. S16** Kelvin probe force microscopy (KPFM) images of perovskite films **a)** without and **b)** with F-PMAI


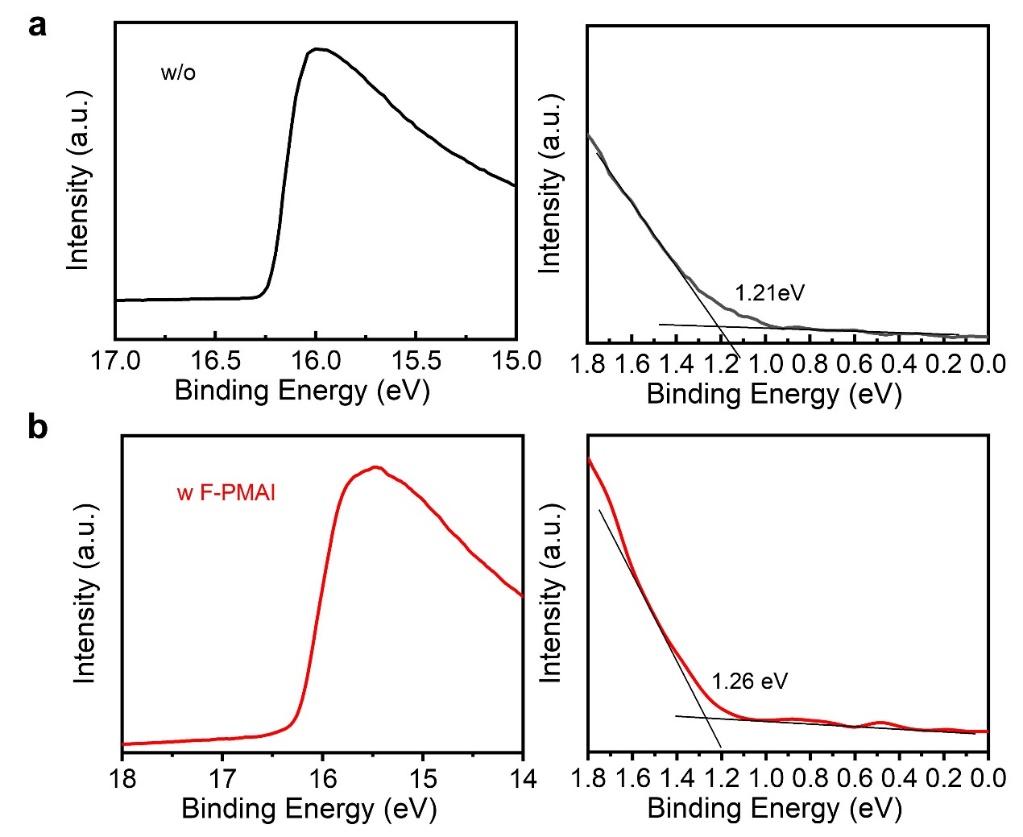


**Fig. S17** Ultraviolet photoelectron spectroscopy (UPS) results of perovskite films without and with F-PMAI


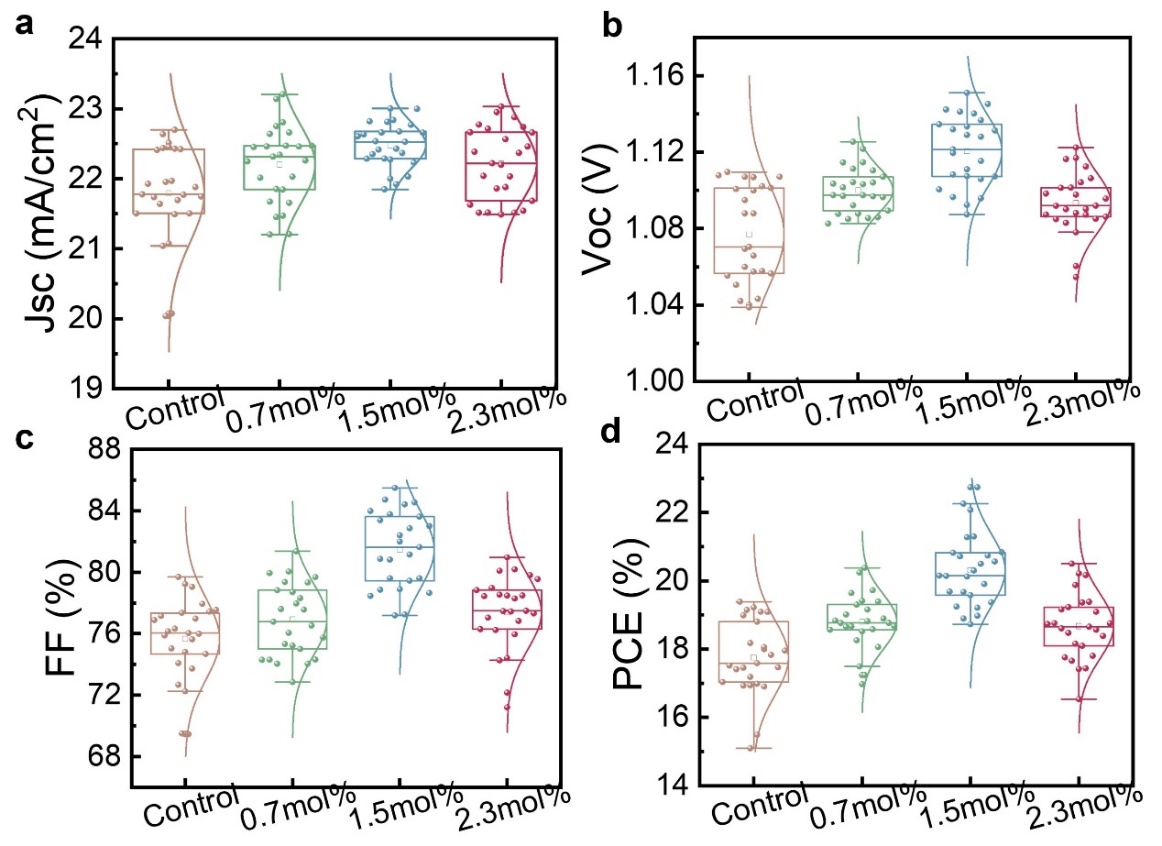


**Fig. S18** **a)** Jsc, **b)** Voc, **c)** FF, **d)** PCE of PV parameters for solar cells with different concentrations of F-PMAI (0, 0.7ml%,1.5mol%, 2.3mol%), 25 devices for each type


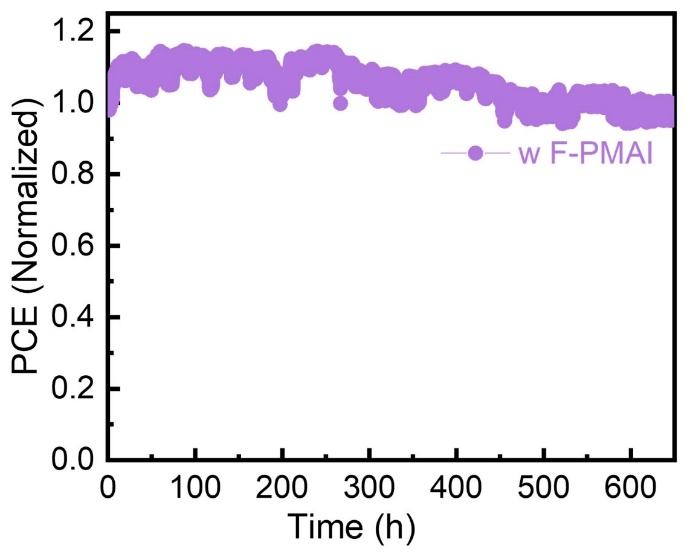


**Fig. S19** Continuous light illumination stability of unencapsulated PSCs with F-PMAI ((100 mW cm^−2^, 25% (RH), 25 °C))


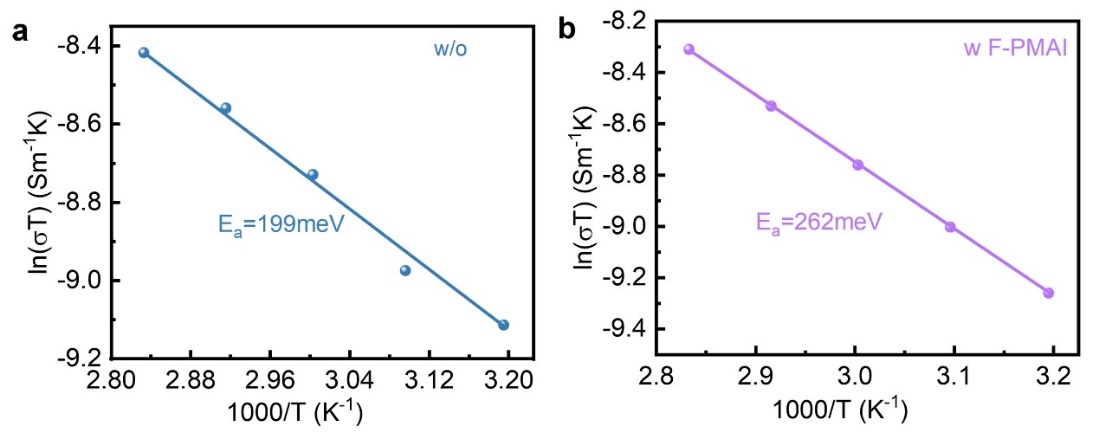


**Fig. S20** Temperature-dependent conductivity of perovskite films **a)** without and **b)** with F-PMAI. Lateral devices with ITO/perovskite/Ag were used


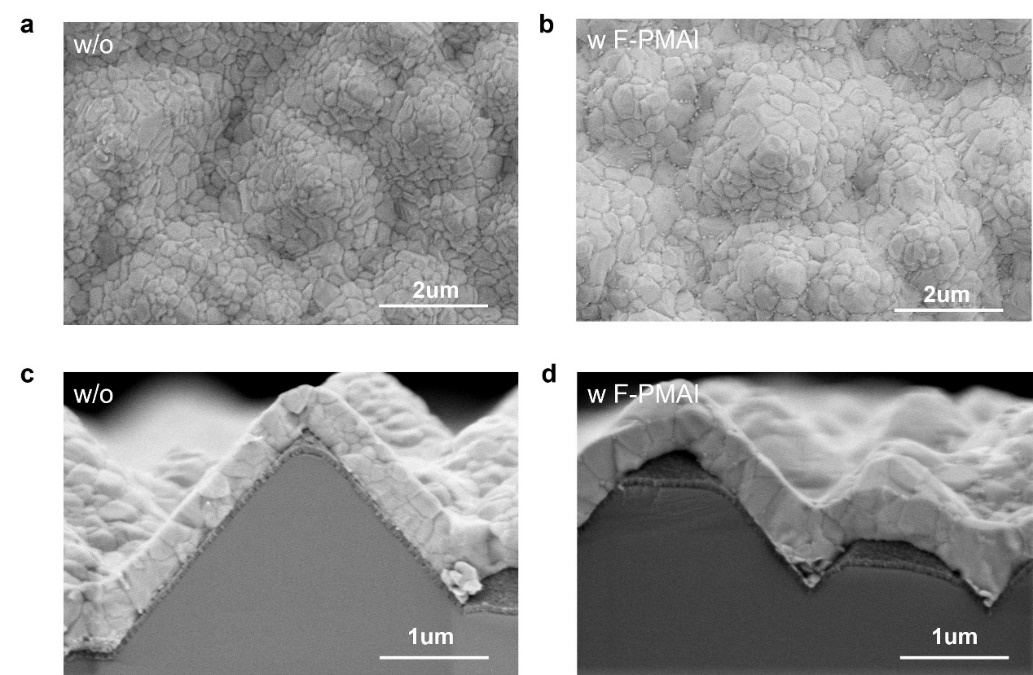


**Fig. S21** Top-view SEM of **a)** without and **b)** with F-PMAI perovskite films on textured silicon substrates. Cross-section SEM of **c)** without and **d)** with F-PMAI perovskite films on textured silicon substrates


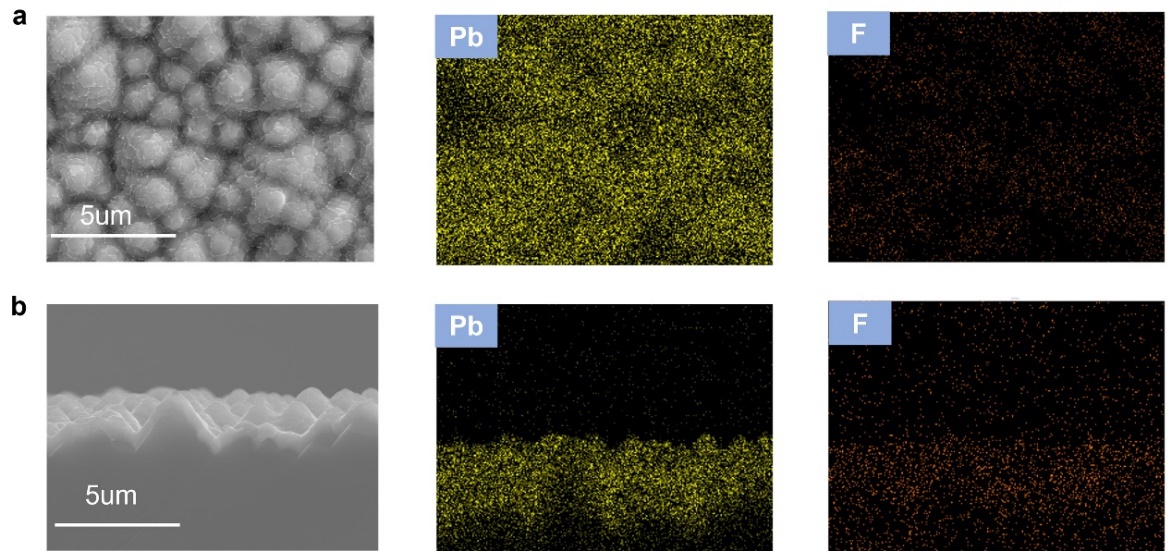


**Fig. S22** **a)** SEM-EDS mapping of perovskite film with F-PMAI on textured silicon substrates. **b)** Cross-sectional SEM-EDS mapping of perovskite film with F-PMAI on textured silicon substrates


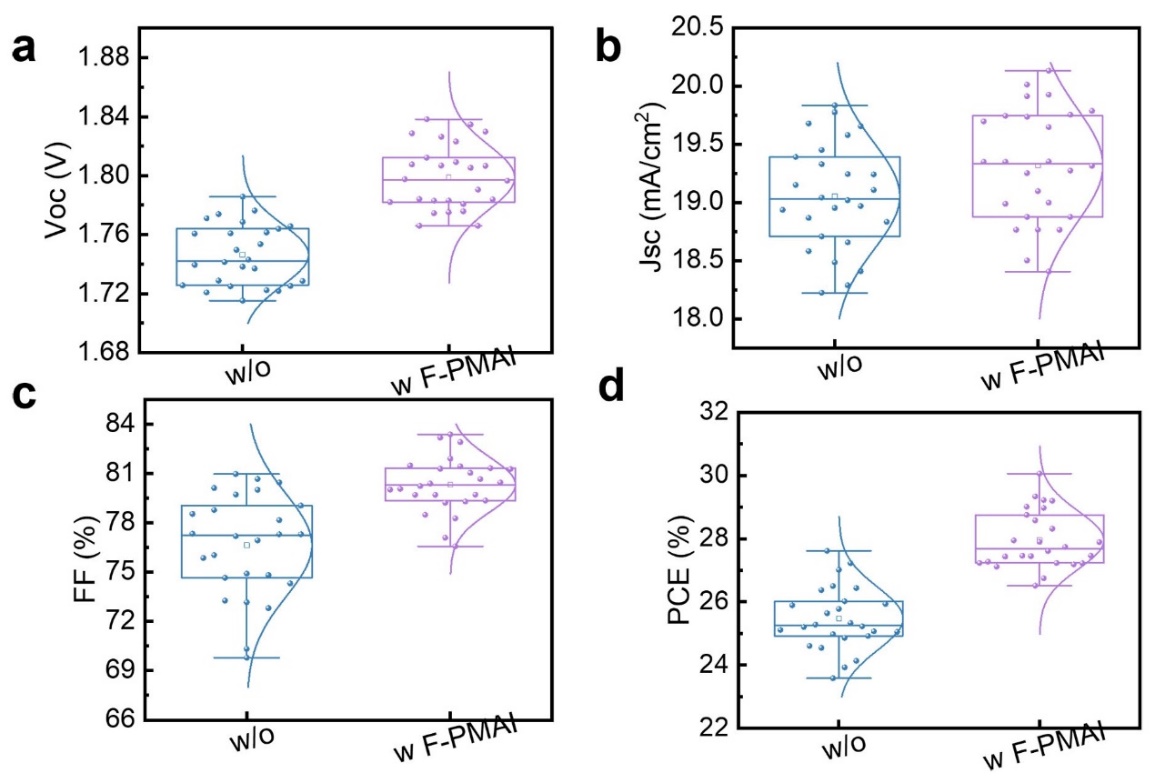


**Fig. S23** Photovoltaic parameters of **a)** V_OC_, **b)** J_SC_, **c)** FF, and **d)** PCE without and with F-PMAI additive derived from 25 tandem devices, respectively

**Table S1** Summary of fitting of the time-resolved photoluminescence (TRPL) of the perovskite films by exponential fitting

| **Sample** | **A_1_ (%)** | $\boldsymbol{\tau}$**_1_ (ns)** | **A_2_ (%)** | $\boldsymbol{\tau}$**_2_ (ns)** | $\boldsymbol{\tau}$**_Av_(ns)** |
| --- | --- | --- | --- | --- | --- |
| w/o | 25.73 | 11.88 | 440.80 | 252.5 | 252.20 |
| w F-PMAI | 5.01 | 142.13 | 94.99 | 541.83 | 536.38 |

**Table S2** Summary of reported monolithic perovskite/silicon tandem solar cells based on micrometer-sized pyramids of textured monocrystalline silicon

| **Institution** | **Tunneling Junction** | **V_OC_**  **(V)** | **J_SC_ (mA/cm^2^)** | **FF**  **(%)** | **PCE**  **(%)** | **SPO**  **(%)** | **E_g_**  **(eV)** | **Area (cm^2^)** | **Refs.** |
| --- | --- | --- | --- | --- | --- | --- | --- | --- | --- |
| EPFL | nc-Si:H(n^+^/p^+^) | 1.78 | 19.5 | 73.1 | 25.5 | 25.2 | 1.60 | 1.42 | [S13] |
| NKU | nc-Si:H(n^+^/p^+^) | 1.808 | 19.78 | 76.9 | 27.48 | / | 1.63 | 0.5091 | [S14] |
| CSEM& EPFL | ITO | 1.91 | 20.47 | 79.8 | 31.25 | / | 1.70 | 1.1677 | [S15] |
| UESTC | ITO | 1.79 | 20.1 | 80.0 | 28.84 | / | 1.65 | 1.2 | [S16] |
| NKU | nc-Si:H(n^+^/p^+^) | 1.85 | 19.4 | 79.6 | 28.5 | 28.2 | 1.68 | 0.5036 | [S17] |
| NJU | ITO | 1.85 | 19.8 | 78.9 | 28.9 | 28.6 | 1.68 | 1.05 | [S18] |
| NCU | ITO | 1.82 | 20.62 | 79.41 | 29.8 | 29.4 | 1.61 | 1 | [S19] |
| NJU | ITO | 1.84 | 20.1 | 77.6 | 28.8 | 28.3 | 1.68 | 1.05 | [S20] |
| **NKU** | **nc-Si:H(n^+^/p^+^)** | **1.81** | **20.01** | **82.91** | **30.05** | **29.4** | **1.60** | **0.5003** | **This work** |

**Supplementary References**

1. G. Kresse, J. Furthmüller, Efficiency of ab-initio total energy calculations for metals and semiconductors using a plane-wave basis set. Comput. Mater. Sci. **6**, 15–50 (1996). <https://doi.org/10.1016/0927-0256(96)00008-0>
2. J.P. Perdew, K. Burke, M. Ernzerhof, Generalized gradient approximation made simple. Phys. Rev. Lett. **77**, 3865–3868 (1996). <https://doi.org/10.1103/physrevlett.77.3865>
3. P.E. Blöchl, Projector augmented-wave method. Phys. Rev. B **50**, 17953–17979 (1994). <https://doi.org/10.1103/physrevb.50.17953>
4. G. Kresse, D. Joubert, From ultrasoft pseudopotentials to the projector augmented-wave method. Phys. Rev. B **59**, 1758–1775 (1999). <https://doi.org/10.1103/physrevb.59.1758>
5. S. Grimme, Semiempirical GGA-type density functional constructed with a long-range dispersion correction. J. Comput. Chem. **27**, 1787–1799 (2006). <https://doi.org/10.1002/jcc.20495>
6. S. Grimme, J. Antony, S. Ehrlich, H. Krieg, A consistent and accurate *ab initio* parametrization of density functional dispersion correction (DFT-D) for the 94 elements H-Pu. J. Chem. Phys. **132**, 154104 (2010). <https://doi.org/10.1063/1.3382344>
7. C. Ma, M.-C. Kang, S.-H. Lee, S.J. Kwon, H.-W. Cha et al., Photovoltaically top-performing perovskite crystal facets. Joule **6**, 2626–2643 (2022). <https://doi.org/10.1016/j.joule.2022.09.012>
8. Y. Lin, Y. Bai, Y. Fang, Q. Wang, Y. Deng et al., Suppressed ion migration in low-dimensional perovskites. ACS Energy Lett. **2**, 1571–1572 (2017). <https://doi.org/10.1021/acsenergylett.7b00442>
9. H. Min, M. Kim, S.-U. Lee, H. Kim, G. Kim et al., Efficient, stable solar cells by using inherent bandgap of a-phase formamidinium lead iodide. Science **366**, 749–753 (2019). <https://doi.org/10.1126/science.aay7044>
10. D. Gao, R. Li, X. Chen, C. Chen, C. Wang et al., Managing interfacial defects and carriers by synergistic modulation of functional groups and spatial conformation for high-performance perovskite photovoltaics based on vacuum flash method. Adv. Mater. **35**, e2301028 (2023). <https://doi.org/10.1002/adma.202301028>
11. S. Xiong, Z. Hou, S. Zou, X. Lu, J. Yang et al., Direct observation on p- to n-type transformation of perovskite surface region during defect passivation driving high photovoltaic efficiency. Joule **5**, 467–480 (2021). <https://doi.org/10.1016/j.joule.2020.12.009>
12. K. Zhang, B. Ding, C. Wang, P. Shi, X. Zhang et al., Highly efficient and stable FAPbI_3_ perovskite solar cells and modules based on exposure of the (011) facet. Nanomicro Lett. **15**, 138 (2023). <https://doi.org/10.1007/s40820-023-01103-8>
13. F. Sahli, J. Werner, B.A. Kamino, M. Bräuninger, R. Monnard et al., Fully textured monolithic perovskite/silicon tandem solar cells with 25.2% power conversion efficiency. Nat. Mater. **17**, 820–826 (2018). <https://doi.org/10.1038/s41563-018-0115-4>
14. Y. Li, B. Shi, Q. Xu, L. Yan, N. Ren et al., Wide bandgap interface layer induced stabilized perovskite/silicon tandem solar cells with stability over ten thousand hours. Adv. Energy Mater. **11**, 2102046 (2021). <https://doi.org/10.1002/aenm.202102046>
15. X.Y. Chin, D. Turkay, J.A. Steele, S. Tabean, S. Eswara et al., Interface passivation for 31.25%-efficient perovskite/silicon tandem solar cells. Science **381**, 59–63 (2023). <https://www.science.org/doi/10.1126/science.adg0091>
16. L. Mao, T. Yang, H. Zhang, J. Shi, Y. Hu et al., Fully textured, production-line compatible monolithic perovskite/silicon tandem solar cells approaching 29% efficiency. Adv. Mater. **34**, e2206193 (2022). <https://doi.org/10.1002/adma.202206193>
17. Q. Xu, B. Shi, Y. Li, L. Yan, W. Duan et al., Conductive passivator for efficient monolithic perovskite/silicon tandem solar cell on commercially textured silicon. Adv. Energy Mater. **12**, 2202404 (2022). <https://doi.org/10.1002/aenm.202202404>
18. X. Luo, H. Luo, H. Li, R. Xia, X. Zheng et al., Efficient perovskite/silicon tandem solar cells on industrially compatible textured silicon. Adv. Mater. **35**, e2207883 (2023). <https://doi.org/10.1002/adma.202207883>
19. F. Zhang, B. Tu, S. Yang, K. Fan, Z. Liu et al., Buried-interface engineering of conformal 2D/3D perovskite heterojunction for efficient perovskite/silicon tandem solar cells on industrially textured silicon. Adv. Mater. **35**, e2303139 (2023). <https://doi.org/10.1002/adma.202303139>
20. H. Luo, X. Zheng, W. Kong, Z. Liu, H. Li et al., Inorganic framework composition engineering for scalable fabrication of perovskite/silicon tandem solar cells. ACS Energy Lett. **8**, 4993–5002 (2023). <https://doi.org/10.1021/acsenergylett.3c02002>
