# Supplementary material for: Textured Perovskite/Silicon Tandem Solar Cells Achieving Over 30% Efficiency Promoted by 4-Fluorobenzylamine Hydroiodide
Source: Nanomicro Lett. 2024 May 2;16:189. doi: 10.1007/s40820-024-01406-4 (PMC11065830; doi:10.1007/s40820-024-01406-4)
Supplement: Supplementary file 2 — Supplementary file2 (PDF 4025 kb) [file 40820_2024_1406_MOESM2_ESM.pdf]

Supporting Information for

Textured Perovskite/Silicon Tandem Solar Cells Achieving Over 30% Efficiency Promoted by 4-Fluorobenzylamine Hydroiodide

Jingjing Liu[1,2,3,4,5], Biao Shi[1,2,3,4,5,*], Qiaojing Xu[1,2,3,4,5], Yucheng Li[1,2,3,4,5], Yuxiang Li[1,2,3,4,5], Pengfei Liu[1,2,3,4,5], Zetong SunLi[1,2,3,4,5], Xuejiao Wang[1,2,3,4,5], Cong Sun[1,2,3,4,5], Wei Han[1,2,3,4,5], Diannan Li[1,2,3,4,5], Sanlong Wang[1,2,3,4,5], Dekun Zhang[1,2,3,4,5], Guangwu Li[6,7], Xiaona Du[1,2,3,4,5], Ying Zhao[1,2,3,4,5], and Xiaodan Zhang[1,2,3,4,5, *]

[1] Institute of Photoelectronic Thin Film Devices and Technology, Renewable Energy Conversion and Storage Center, Solar Energy Conversion Center, Nankai University, Tianjin 300350, P. R. China

[2] Key Laboratory of Photoelectronic Thin Film Devices and Technology of Tianjin, Tianjin 300350, P. R. China

[3] Haihe Laboratory of Sustainable Chemical Transformations, Tianjin 300192, P. R. China

[4] Engineering Research Center of Thin Film Photoelectronic Technology of Ministry of Education, Tianjin 300350, P. R. China

[5] Collaborative Innovation Center of Chemical Science and Engineering (Tianjin), Tianjin 300072, P. R. China

[6] Center of Single-Molecule Sciences, Institute of Modern Optics, Tianjin Key Laboratory of Micro-Scale Optical Information Science and Technology, College of Electronic Information and Optical Engineering, Nankai University, 38 Tongyan Road, Jinnan District, Tianjin 300350, P. R. China

[7] Shenzhen Research Institute of Nankai University, 16th Floor, Yantian Science & Technology Building, Haishan Street, Yantian District, Shenzhen, 518083, P. R. China

*Corresponding authors. E-mail: biaos_xiaog@163.com (Biao Shi), xdzhang@nankai.edu.cn (Xiaodan Zhang)

S1 Calculation Methods

S1.1 Simulation Calculation

First-principle calculations were performed by density functional theory (DFT) using the Vienna Ab-initio Simulation Package (VASP) package [S1]. The generalized gradient approximation (GGA) with the Perdew-Burke-Ernzerhof (PBE) functional was used to describe the electronic exchange and correlation effects [S2–S4]. Uniform G-centered k-point meshes with a resolution of $2\pi\times0.05$ Å^{-1} and Methfessel-Paxton electronic smearing were adopted for the integration in the Brillouin zone for geometric optimization. The simulation was run with a cutoff energy of 500 eV throughout the computations. These settings ensure convergence of the total energies to within 1 meV per atom. Structure relaxation proceeded until all forces on atoms were less than 10 meV Å^{-1} and the total stress tensor was within 0.03 GPa of the target value. The DFT-D2 Van der Walls correction by Grimmie [S5, S6] was also considered in all

calculations.

The adsorption energies of F-PMAI molecule adsorbed on FA/MA/Cs-PbI_3 (100) and (111) surfaces were calculated by the following equation: $\Delta E_{(ads)}=E_{(total)}-E_{(surface)}-E_{(F\text{-}PMAI)}$, where $E_{(total)}$ is the energy of F-PMAI molecule adsorbed on FA/MA/Cs-PbI_3 (100) and (111) surfaces, $E_{(surface)}$ is the energy of FA/MA/Cs-PbI_3 (100) and (111) surfaces, and $E_{(F\text{-}PMAI)}$ is the energy of the F-PMAI molecule. The more negative the value, the stronger the binding ability.

S1.2 Ion Migration Activation Energy (E_a) Calculation

Temperature-dependent conductivity, σ (T), was measured to compare the activation energy (E_a), for ion migration. E_a can be calculated from Equation [S7, S8] $\sigma(T)T = \sigma_0 e^{(-\frac{E_a}{KT})}$,Where k is the Boltzmann's constant, σ_0 is the constant, and T is the temperature. Based on $\ln(\sigma(T)T)$versus1000/T plots, the Ea for ion migration was extracted from the slope of the fitted lines at relatively higher temperature.

S1.3 Space Charge Limited Current (SCLC) Measurements

The trap densities were extracted using the equation: $N_t = \frac{2\varepsilon\varepsilon_0 V_{TFL}}{eL^2}$, where N_t denotes the trap state density, ε and ε_0 are the relative dielectric constant and the vacuum dielectric constant, respectively, V_{TFL} is the trap-filled limit voltage, e is the electron charge and L is the thickness of perovskite film [S9].

S1.4 Capacitance-voltage (C-V) Measurements

The Mott-Schottky equation: $\frac{1}{C^2} = \frac{2(V_{bi}-V)}{A^2 e\varepsilon\varepsilon_0 N_A}$ (A is the device area, ε and ε_0 are the relative and vacuum permittivity, and N_A is carrier concentration) [S10].

S1.5 Dark *J-V* Curves Measurement

In the dark *J-V* curves, the ideal factor (m) is extracted from the equation: $m = (\frac{KT}{q}\frac{dlnJ}{dV})^{-1}$. When m=1, bimolecular recombination dominates; when m=2, trap-assisted recombination dominates [S11].

S1.6 Light Intensity-dependent V_{oc} Measurement

The slope of the dependence of V_{oc} verse light intensity (I) is used to evaluate the degree of the trap-assisted recombination via the equation $V_{oc} = \frac{nKTln(I)}{q} + c$ (n is the ideal factor, K is the Boltzmann constant, T is the absolute temperature, I is the incident light intensity, q is the elementary charge, and c is the constant) from the Shockley−Read−Hall recombination mechanism. The closer the value of n to 1, the less trap-assisted nonradiative recombination exists in the PSCs [S12].

S2 Supplementary Figures

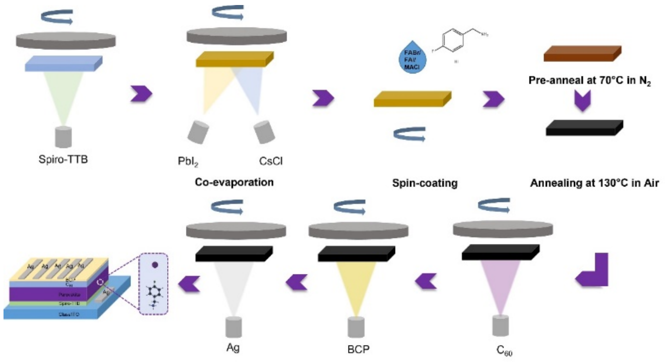

Fig. S1 Schematic of the hybrid two-step deposition perovskite solar cells

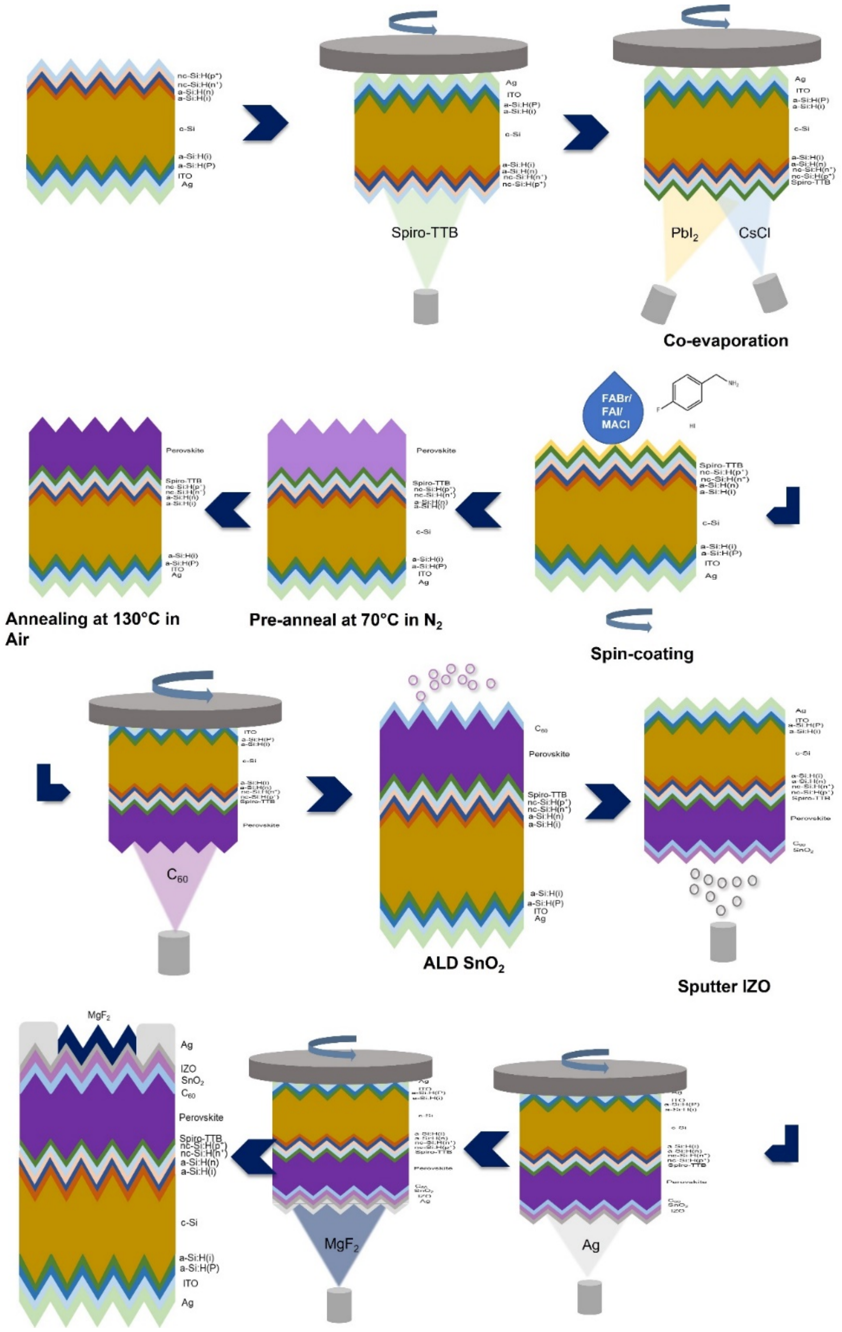

Fig. S2 Schematic of the fabrication of perovskite/silicon tandem solar cells

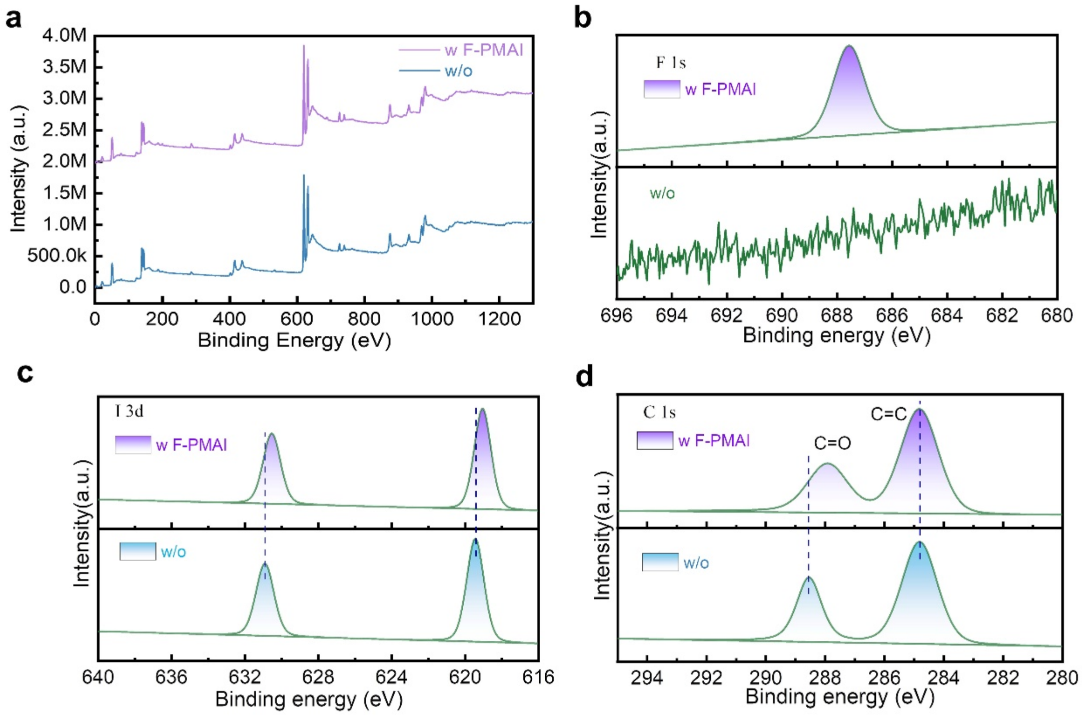

Fig. S3 a) Full-scale X-ray photoelectron spectroscopy (XPS) spectrum of perovskite films without and with F-PMAI. XPS spectra of **b)** F 1s, **c)** I 3d and **d)** C 1s of perovskite films without and with F-PMAI additive

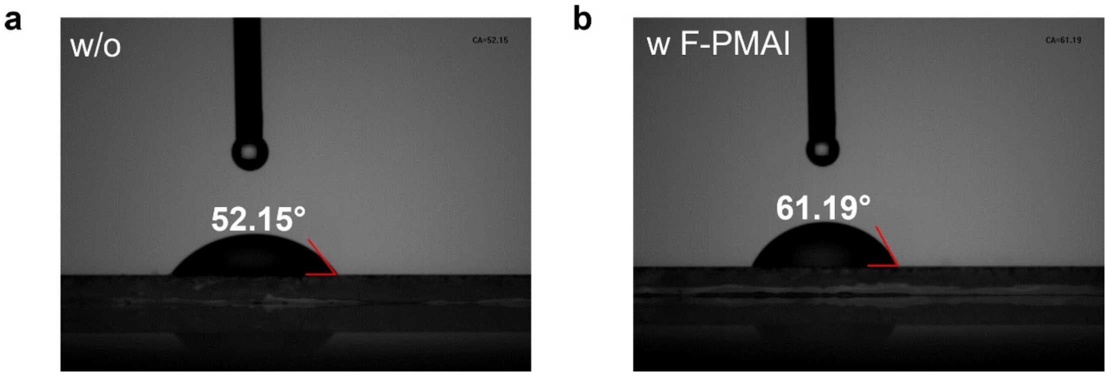

Fig. S4 Images of water droplets on the surface of perovskite films **a)** without and **b)** with F-PMAI

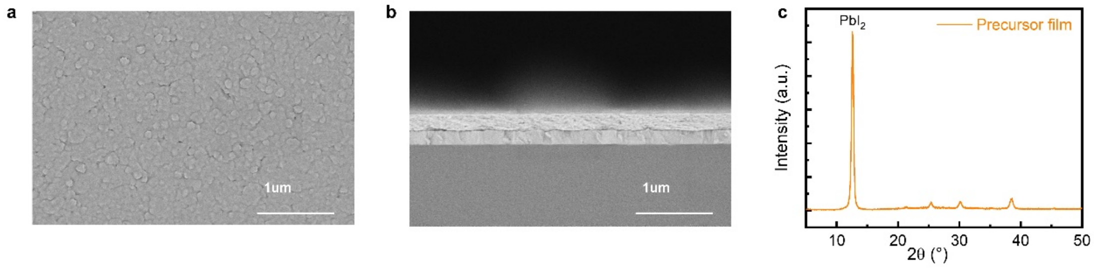

Fig. S5 Top-view **a)** and cross-sectional **b)** SEM images of co-evaporated precursor films. **c)** XRD spectra of co-evaporated precursor films

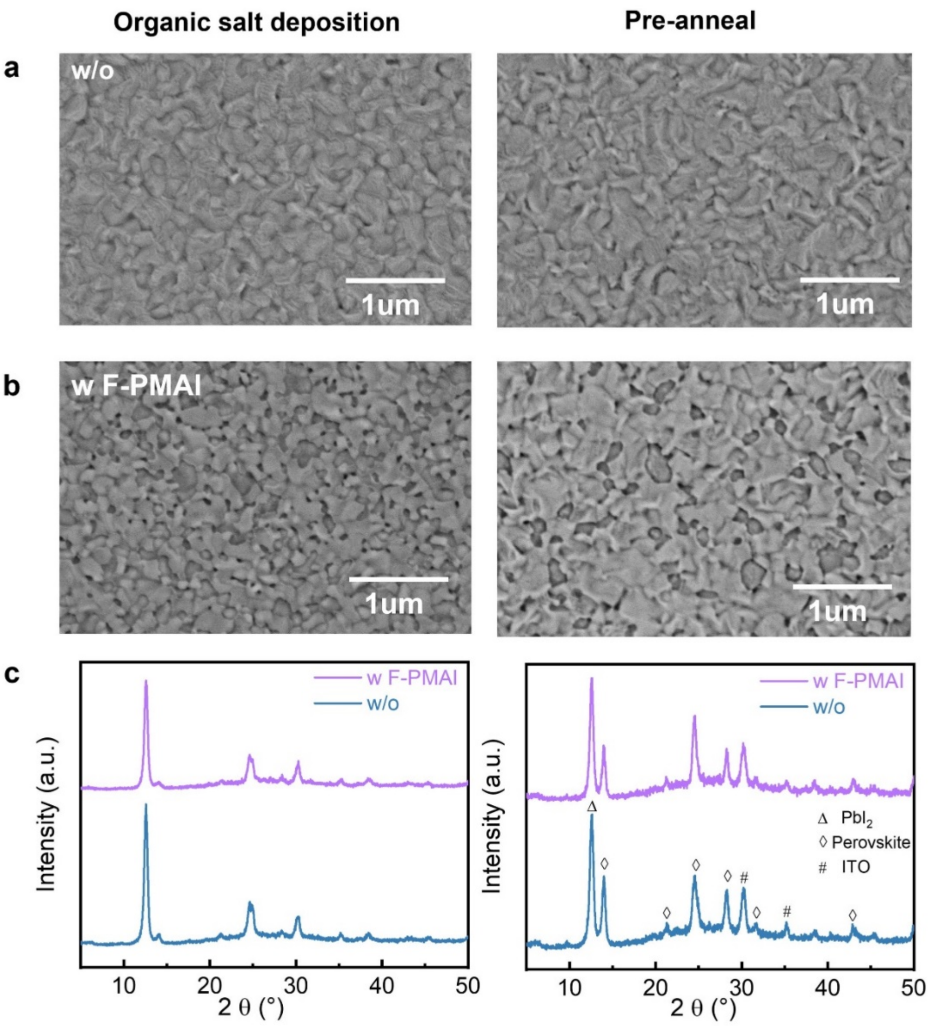

Fig. S6 Top-view and SEM images of perovskite films **a)** without and **b)** with F-PMAI during the different processes. **c)** XRD patterns of perovskite films without and with F-PMAI after different processes, including organic salt deposition and pre-annealing

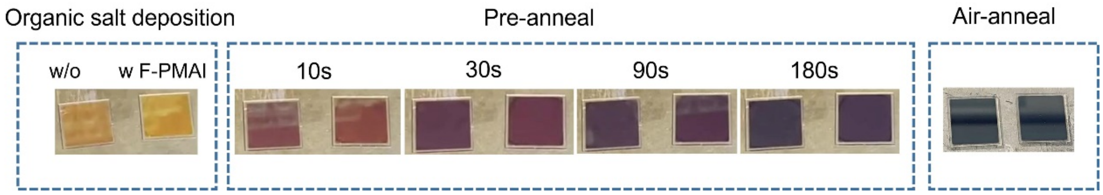

Fig. S7 Photographs of perovskite thin films without and with additives at different processes and time

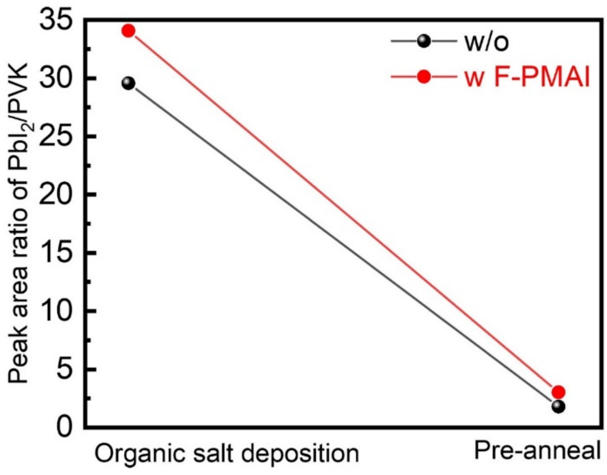

Fig. S8 The peak intensity ratio of PbI_2/PVK at the organic salt deposition and pre-anneal stage respectively

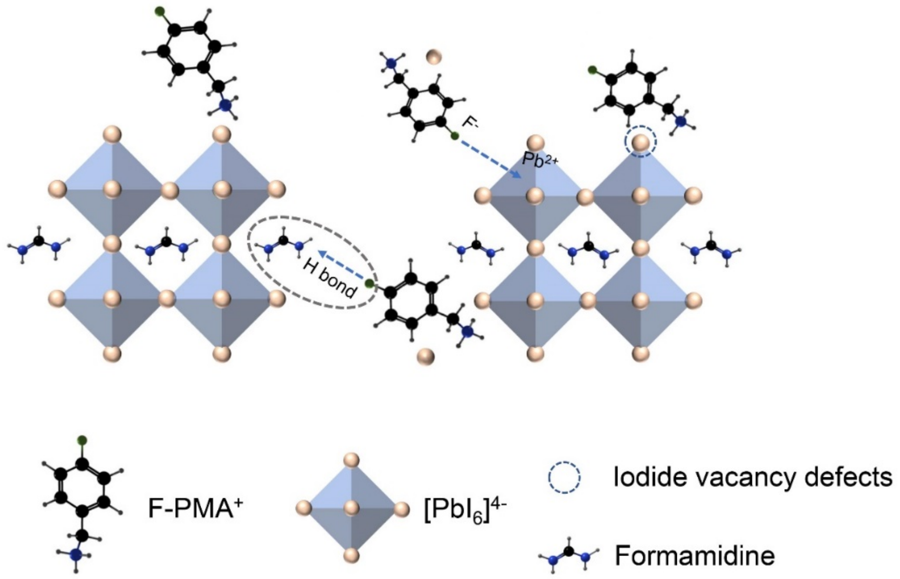

Fig. S9 Schematic diagram of interaction mechanism of the F-PMAI on perovskite

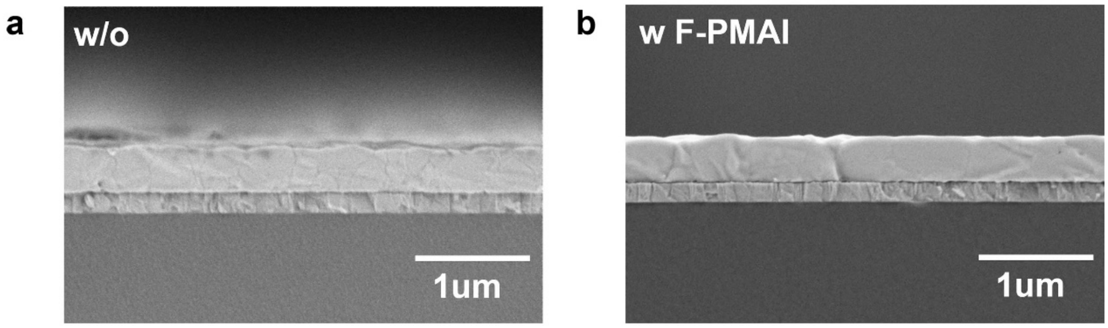

Fig. S10 Cross-section SEM images of perovskite films **a)** without and **b)** with F-PMAI

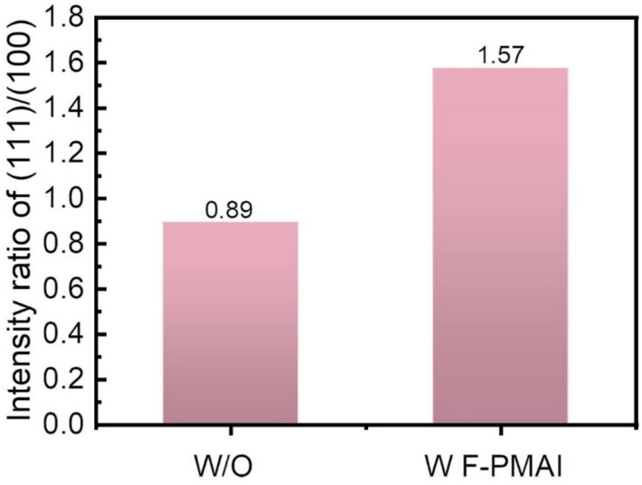

Fig. S11 Peak intensity ratio of (111) and (100) perovskite in Fig. 1d

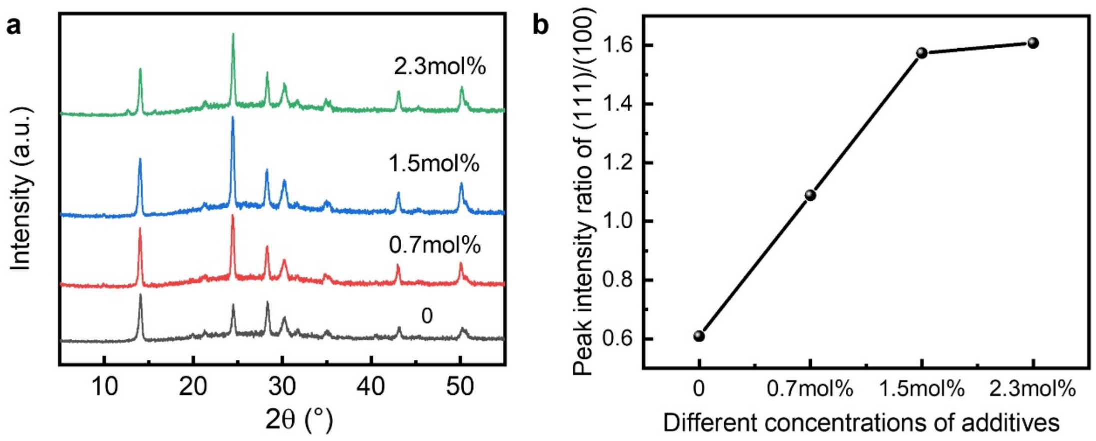

Fig. S12 a) XRD pattern of perovskite film with different F-PMAI concentrations (0, 0.7mol%, 1.5mol%, 2.3mol%). **b)** Peak intensity ratio of (111) and (100) perovskite

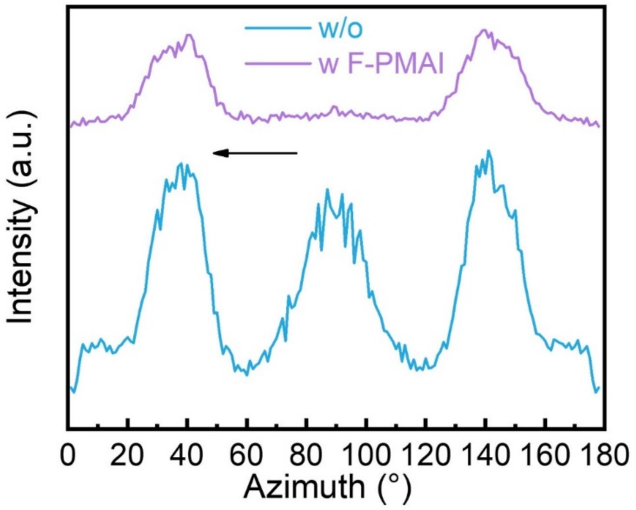

Fig. S13 Integrated GIWAXS intensity plots azimuthally along the ring at a q≈10 nm^{-1}, assigned to the (100) plane of perovskite films without and with F-PMAI

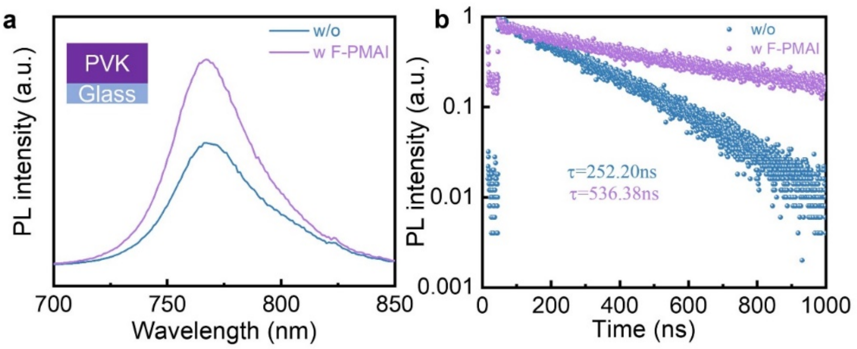

Fig. S14 a) PL and **b)** TRPL spectra of perovskite films without and with F-PMAI deposited on ITO substrates

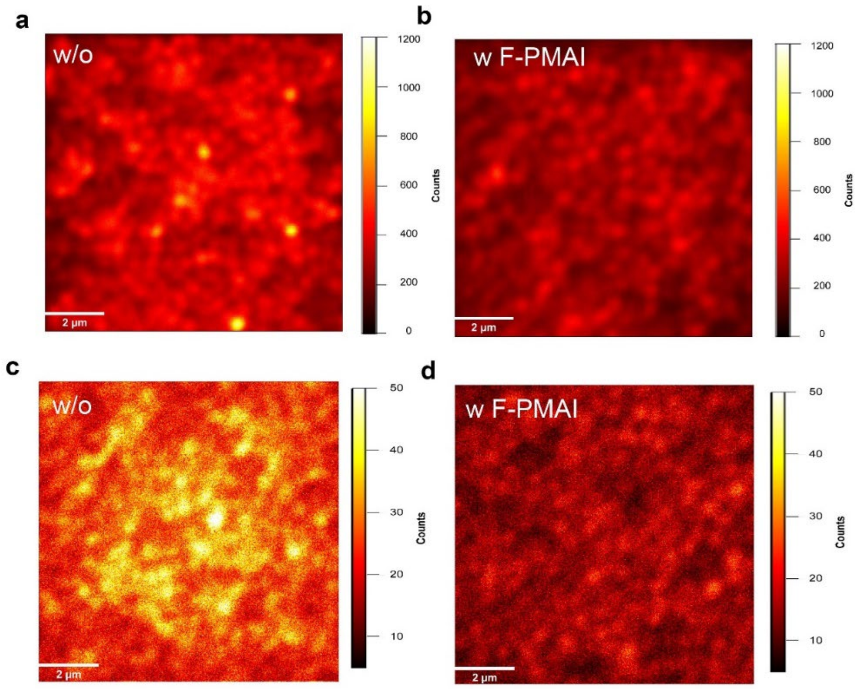

Fig. S15 PL mapping of perovskite films **a)** without and **b)** with F-PMAI with a structure ITO /Spiro-TTB /perovskite. PL mapping of perovskite films **c)** without and **d)** with F-PMAI with a structure of ITO/perovskite/C_{60}

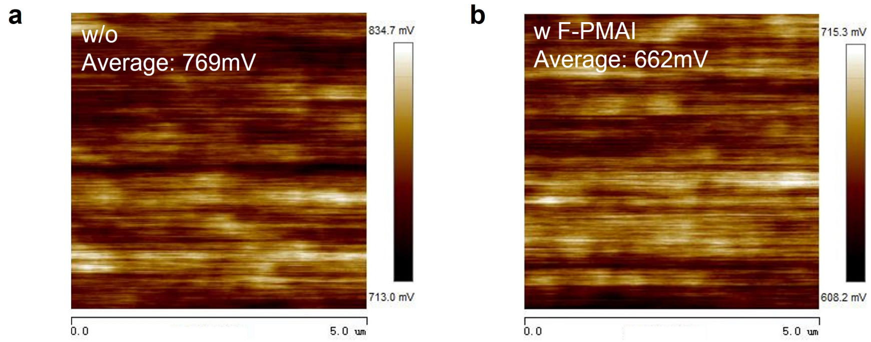

Fig. S16 Kelvin probe force microscopy (KPFM) images of perovskite films **a)** without and **b)** with F-PMAI

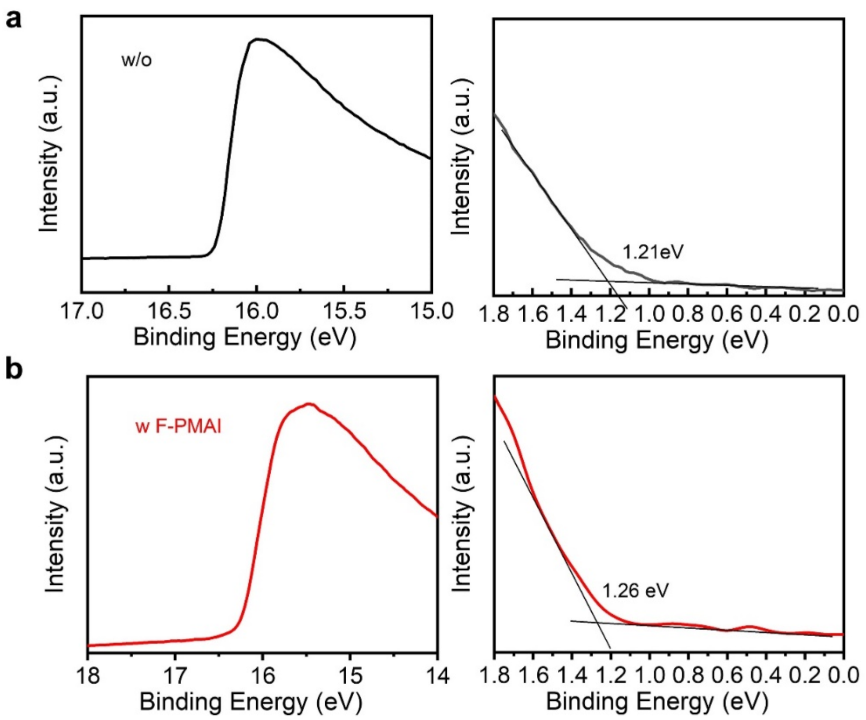

Fig. S17 Ultraviolet photoelectron spectroscopy (UPS) results of perovskite films without and with F-PMAI

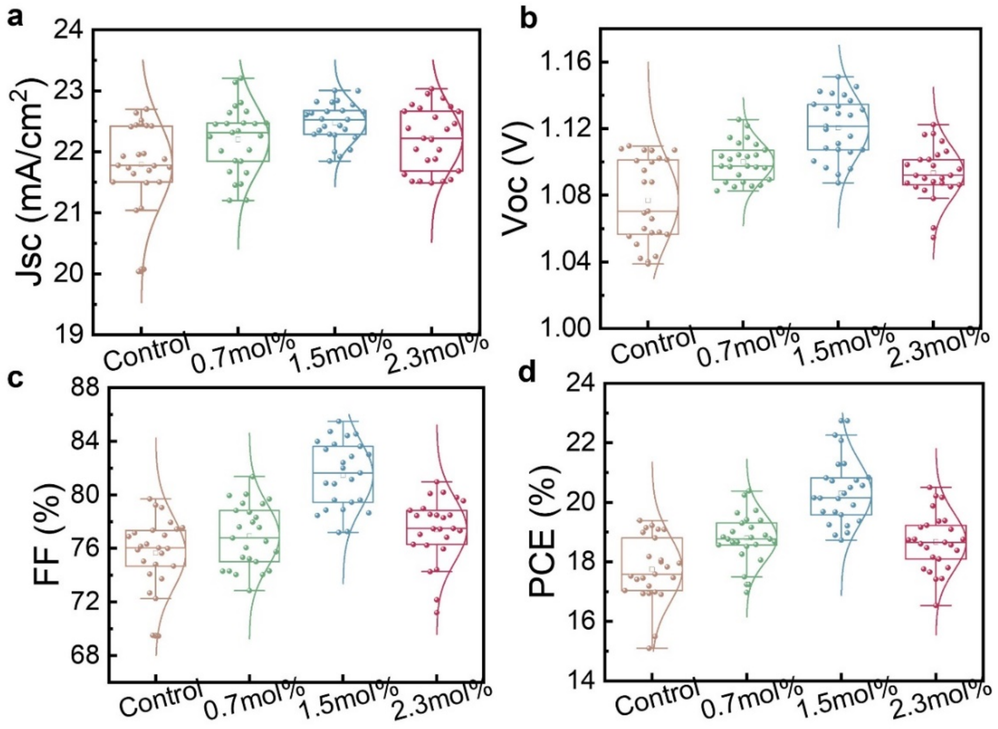

Fig. S18 a) Jsc, **b)** Voc, **c)** FF, **d)** PCE of PV parameters for solar cells with different concentrations of F-PMAI (0, 0.7ml%,1.5mol%, 2.3mol%), 25 devices for each type

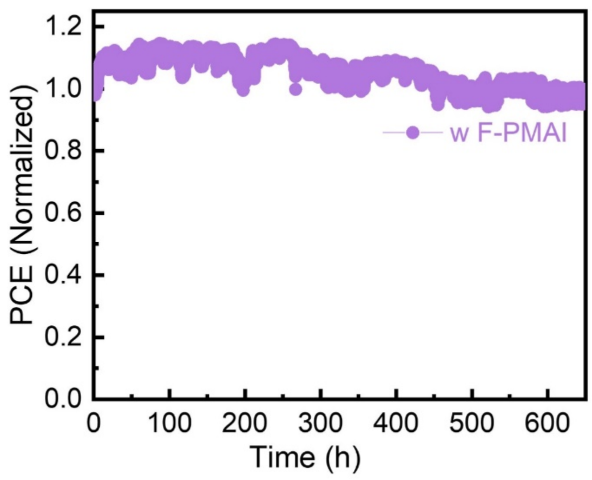

Fig. S19 Continuous light illumination stability of unencapsulated PSCs with F-PMAI ((100 mW cm^{-2}, 25% (RH), 25 °C))

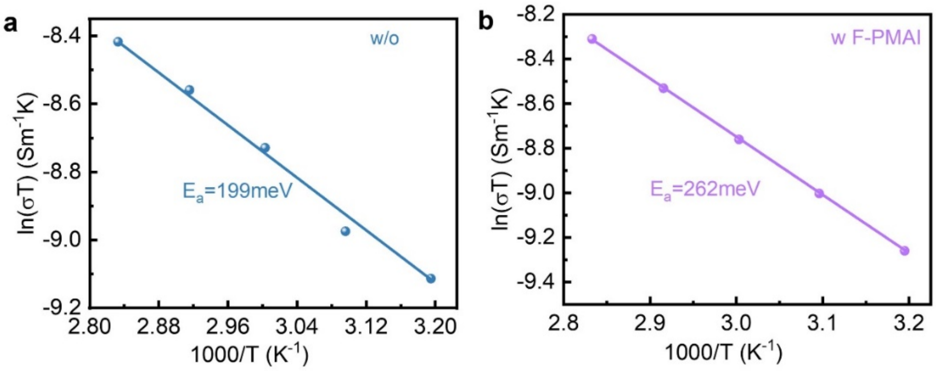

Fig. S20 Temperature-dependent conductivity of perovskite films **a)** without and **b)** with F-PMAI. Lateral devices with ITO/perovskite/Ag were used

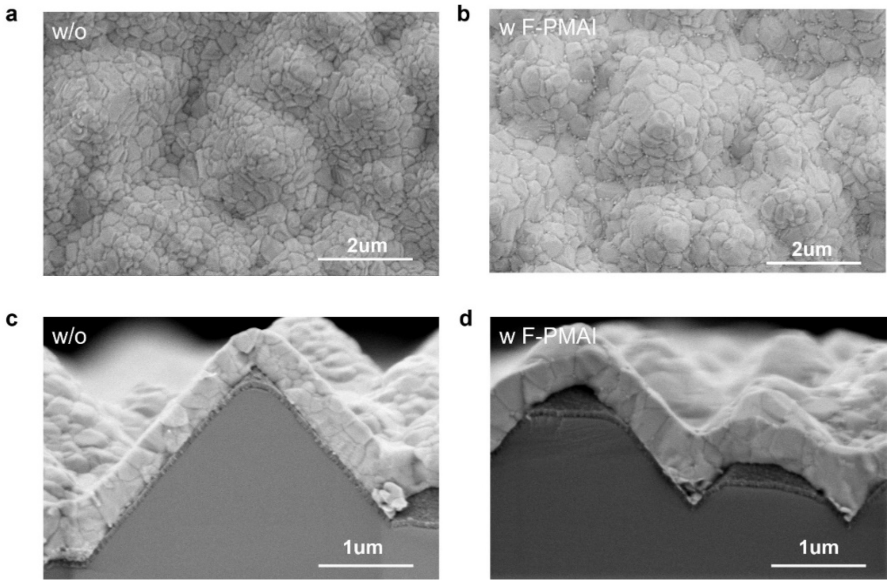

Fig. S21 Top-view SEM of **a)** without and **b)** with F-PMAI perovskite films on textured silicon substrates. Cross-section SEM of **c)** without and **d)** with F-PMAI perovskite films on textured silicon substrates

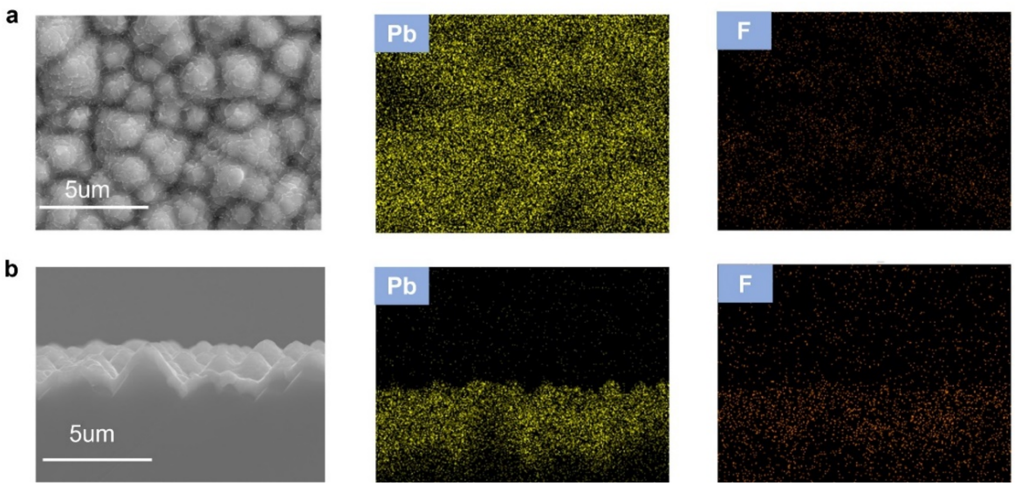

Fig. S22 a) SEM-EDS mapping of perovskite film with F-PMAI on textured silicon substrates. **b)** Cross-sectional SEM-EDS mapping of perovskite film with F-PMAI on textured silicon substrates

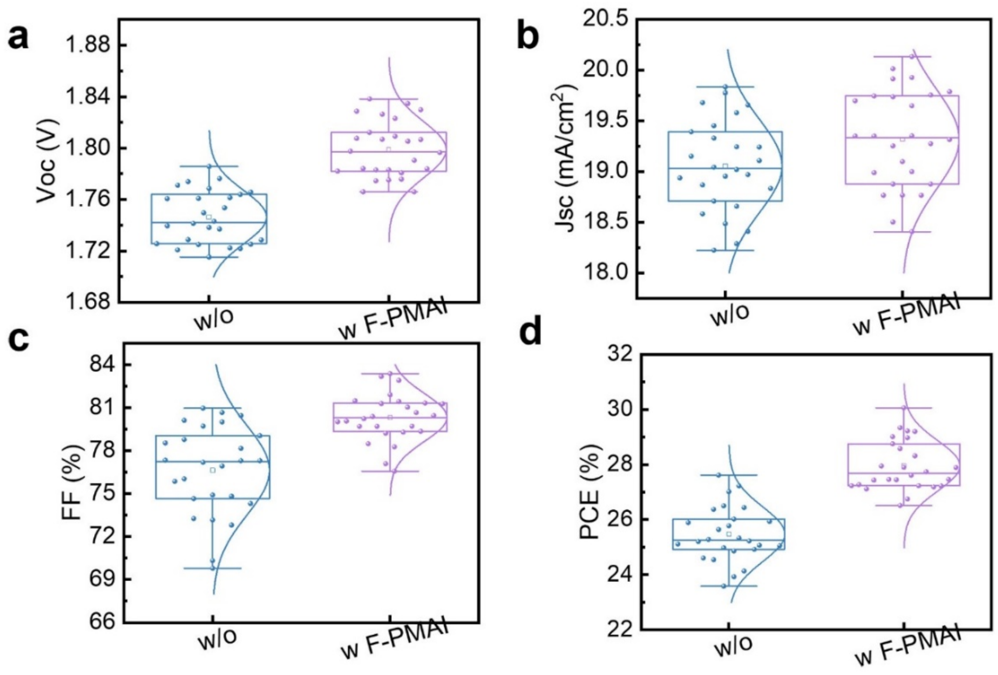

Fig. S23 Photovoltaic parameters of **a)** V_{OC}, **b)** J_{SC}, **c)** FF, and **d)** PCE without and with F-PMAI additive derived from 25 tandem devices, respectively

Table S1 Summary of fitting of the time-resolved photoluminescence (TRPL) of the perovskite films by exponential fitting

Sample	A_1 (%)	τ_1 (ns)	A_2 (%)	τ_2 (ns)	τ_{Av} (ns)
w/o	25.73	11.88	440.80	252.5	252.20
w F-PMAI	5.01	142.13	94.99	541.83	536.38

Table S2 Summary of reported monolithic perovskite/silicon tandem solar cells based on micrometer-sized pyramids of textured monocrystalline silicon

Institution	Tunneling Junction	V_{OC} (V)	J_{SC} (mA/cm^2)	FF (%)	PCE (%)	SPO (%)	E_g (eV)	Area (cm^2)	Refs.
EPFL	nc-Si:H(n^+/p^+)	1.78	19.5	73.1	25.5	25.2	1.60	1.42	[S13]
NKU	nc-Si:H(n^+/p^+)	1.808	19.78	76.9	27.48	/	1.63	0.5091	[S14]
CSEM& EPFL	ITO	1.91	20.47	79.8	31.25	/	1.70	1.1677	[S15]
UESTC	ITO	1.79	20.1	80.0	28.84	/	1.65	1.2	[S16]
NKU	nc-Si:H(n^+/p^+)	1.85	19.4	79.6	28.5	28.2	1.68	0.5036	[S17]
NJU	ITO	1.85	19.8	78.9	28.9	28.6	1.68	1.05	[S18]
NCU	ITO	1.82	20.62	79.41	29.8	29.4	1.61	1	[S19]
NJU	ITO	1.84	20.1	77.6	28.8	28.3	1.68	1.05	[S20]
**NKU**	**nc-Si:H(n^+/p^+)**	**1.81**	**20.01**	**82.91**	**30.05**	**29.4**	**1.60**	**0.5003**	**This work**

Supplementary References

[S1] G. Kresse, J. Furthmüller, Efficiency of ab-initio total energy calculations for metals and semiconductors using a plane-wave basis set. Comput. Mater. Sci. **6**, 15–50 (1996). https://doi.org/10.1016/0927-0256(96)00008-0

[S2] J.P. Perdew, K. Burke, M. Ernzerhof, Generalized gradient approximation made simple. Phys. Rev. Lett. **77**, 3865–3868 (1996). https://doi.org/10.1103/physrevlett.77.3865

[S3] P.E. Blöchl, Projector augmented-wave method. Phys. Rev. B **50**, 17953–17979 (1994). https://doi.org/10.1103/physrevb.50.17953

[S4] G. Kresse, D. Joubert, From ultrasoft pseudopotentials to the projector augmented-wave method. Phys. Rev. B **59**, 1758–1775 (1999). https://doi.org/10.1103/physrevb.59.1758

[S5] S. Grimme, Semiempirical GGA-type density functional constructed with a long-range dispersion correction. J. Comput. Chem. **27**, 1787–1799 (2006). https://doi.org/10.1002/jcc.20495

[S6] S. Grimme, J. Antony, S. Ehrlich, H. Krieg, A consistent and accurate *ab initio* parametrization of density functional dispersion correction (DFT-D) for the 94 elements H-Pu. J. Chem. Phys. **132**, 154104 (2010). https://doi.org/10.1063/1.3382344

[S7] C. Ma, M.-C. Kang, S.-H. Lee, S.J. Kwon, H.-W. Cha et al., Photovoltaically top-

performing perovskite crystal facets. Joule **6**, 2626–2643 (2022). https://doi.org/10.1016/j.joule.2022.09.012

[S8] Y. Lin, Y. Bai, Y. Fang, Q. Wang, Y. Deng et al., Suppressed ion migration in low-dimensional perovskites. ACS Energy Lett. **2**, 1571–1572 (2017). https://doi.org/10.1021/acsenergylett.7b00442

[S9] H. Min, M. Kim, S.-U. Lee, H. Kim, G. Kim et al., Efficient, stable solar cells by using inherent bandgap of a-phase formamidinium lead iodide. Science **366**, 749–753 (2019). https://doi.org/10.1126/science.aay7044

[S10] D. Gao, R. Li, X. Chen, C. Chen, C. Wang et al., Managing interfacial defects and carriers by synergistic modulation of functional groups and spatial conformation for high-performance perovskite photovoltaics based on vacuum flash method. Adv. Mater. **35**, e2301028 (2023). https://doi.org/10.1002/adma.202301028

[S11] S. Xiong, Z. Hou, S. Zou, X. Lu, J. Yang et al., Direct observation on p- to n-type transformation of perovskite surface region during defect passivation driving high photovoltaic efficiency. Joule **5**, 467–480 (2021). https://doi.org/10.1016/j.joule.2020.12.009

[S12] K. Zhang, B. Ding, C. Wang, P. Shi, X. Zhang et al., Highly efficient and stable $FAPbI_3$ perovskite solar cells and modules based on exposure of the (011) facet. Nanomicro Lett. **15**, 138 (2023). https://doi.org/10.1007/s40820-023-01103-8

[S13] F. Sahli, J. Werner, B.A. Kamino, M. Bräuninger, R. Monnard et al., Fully textured monolithic perovskite/silicon tandem solar cells with 25.2% power conversion efficiency. Nat. Mater. **17**, 820–826 (2018). https://doi.org/10.1038/s41563-018-0115-4

[S14] Y. Li, B. Shi, Q. Xu, L. Yan, N. Ren et al., Wide bandgap interface layer induced stabilized perovskite/silicon tandem solar cells with stability over ten thousand hours. Adv. Energy Mater. **11**, 2102046 (2021). https://doi.org/10.1002/aenm.202102046

[S15] X.Y. Chin, D. Turkay, J.A. Steele, S. Tabean, S. Eswara et al., Interface passivation for 31.25%-efficient perovskite/silicon tandem solar cells. Science **381**, 59–63 (2023). https://www.science.org/doi/10.1126/science.adg0091

[S16] L. Mao, T. Yang, H. Zhang, J. Shi, Y. Hu et al., Fully textured, production-line compatible monolithic perovskite/silicon tandem solar cells approaching 29% efficiency. Adv. Mater. **34**, e2206193 (2022). https://doi.org/10.1002/adma.202206193

[S17] Q. Xu, B. Shi, Y. Li, L. Yan, W. Duan et al., Conductive passivator for efficient monolithic perovskite/silicon tandem solar cell on commercially textured silicon. Adv. Energy Mater. **12**, 2202404 (2022). https://doi.org/10.1002/aenm.202202404

[S18] X. Luo, H. Luo, H. Li, R. Xia, X. Zheng et al., Efficient perovskite/silicon tandem solar cells on industrially compatible textured silicon. Adv. Mater. **35**, e2207883 (2023). https://doi.org/10.1002/adma.202207883

[S19] F. Zhang, B. Tu, S. Yang, K. Fan, Z. Liu et al., Buried-interface engineering of conformal 2D/3D perovskite heterojunction for efficient perovskite/silicon tandem solar cells on industrially textured silicon. Adv. Mater. **35**, e2303139 (2023). https://doi.org/10.1002/adma.202303139

[S20] H. Luo, X. Zheng, W. Kong, Z. Liu, H. Li et al., Inorganic framework composition engineering for scalable fabrication of perovskite/silicon tandem solar cells. ACS Energy Lett. **8**, 4993–5002 (2023). https://doi.org/10.1021/acsenergylett.3c02002
